# A systematic review of research on augmentative and alternative communication brain-computer interface systems for individuals with disabilities

**DOI:** 10.3389/fnhum.2022.952380

**Published:** 2022-07-27

**Authors:** Betts Peters, Brandon Eddy, Deirdre Galvin-McLaughlin, Gail Betz, Barry Oken, Melanie Fried-Oken

**Affiliations:** ^1^Consortium for Accessible Multimodal Brain-Body Interfaces, United States; ^2^REKNEW Projects, Institute on Development and Disability, Department of Pediatrics, Oregon Health and Science University, Portland, OR, United States; ^3^Speech and Hearing Sciences Department, Portland State University, Portland, OR, United States; ^4^Health Sciences and Human Services Library, University of Maryland, Baltimore, MD, United States; ^5^Department of Neurology, Oregon Health & Science University, Portland, OR, United States

**Keywords:** augmentative and alternative communication (AAC), brain-computer interface (BCI), locked-in syndrome (LIS), dysarthria, tetraplegia, systematic review

## Abstract

Augmentative and alternative communication brain-computer interface (AAC-BCI) systems are intended to offer communication access to people with severe speech and physical impairment (SSPI) without requiring volitional movement. As the field moves toward clinical implementation of AAC-BCI systems, research involving participants with SSPI is essential. Research has demonstrated variability in AAC-BCI system performance across users, and mixed results for comparisons of performance for users with and without disabilities. The aims of this systematic review were to (1) describe study, system, and participant characteristics reported in BCI research, (2) summarize the communication task performance of participants with disabilities using AAC-BCI systems, and (3) explore any differences in performance for participants with and without disabilities. Electronic databases were searched in May, 2018, and March, 2021, identifying 6065 records, of which 73 met inclusion criteria. Non-experimental study designs were common and sample sizes were typically small, with approximately half of studies involving five or fewer participants with disabilities. There was considerable variability in participant characteristics, and in how those characteristics were reported. Over 60% of studies reported an average selection accuracy ≤70% for participants with disabilities in at least one tested condition. However, some studies excluded participants who did not reach a specific system performance criterion, and others did not state whether any participants were excluded based on performance. Twenty-nine studies included participants both with and without disabilities, but few reported statistical analyses comparing performance between the two groups. Results suggest that AAC-BCI systems show promise for supporting communication for people with SSPI, but they remain ineffective for some individuals. The lack of standards in reporting outcome measures makes it difficult to synthesize data across studies. Further research is needed to demonstrate efficacy of AAC-BCI systems for people who experience SSPI of varying etiologies and severity levels, and these individuals should be included in system design and testing. Consensus in terminology and consistent participant, protocol, and performance description will facilitate the exploration of user and system characteristics that positively or negatively affect AAC-BCI use, and support innovations that will make this technology more useful to a broader group of people.

**Clinical trial registration:**

https://www.crd.york.ac.uk/prospero/display_record.php?ID=CRD42018095345, PROSPERO: CRD42018095345.

## 1. Introduction

Brain-computer interface (BCI) systems are designed to record neurophysiological signals, extract signal features, and output information to a computer (Wolpaw and Wolpaw, 2012). One promising application for BCI technology is the control of augmentative and alternative communication (AAC) devices, computer-based devices used for communication by individuals with speech or language impairments. Because AAC-BCI systems can be operated without reliable volitional movement, they offer a potential means of communication access for people with locked-in syndrome (LIS) or other forms of severe speech and physical impairment (SSPI) who may have difficulty using other AAC access methods (e.g., eye gaze, switch access, touch access) (Akcakaya et al., 2013; Rezeika et al., 2018; Pitt et al., 2019). Researchers have explored the use of AAC-BCI systems by individuals with a variety of medical conditions that may cause SSPI, including amyotrophic lateral sclerosis (ALS) (Kübler et al., 1999; Wolpaw et al., 2018; Medina-Juliá et al., 2020; Miao et al., 2020; Peters et al., 2020), muscular dystrophy (Halder et al., 2016a), stroke (Kaufmann et al., 2013a; Kleih et al., 2016), traumatic brain injury (Combaz et al., 2013; Lesenfants et al., 2014), cerebral palsy (Käthner et al., 2017), and spinal muscular atrophy (Zickler et al., 2011). Many participants in these studies presented with classic LIS (in which only eye movement and blinking are preserved) or incomplete LIS (in which small movements of other body parts are possible) (Bauer et al., 1979; Smith and Delargy, 2005). Few studies have reported successful AAC-BCI system use by individuals with total LIS, who demonstrate no voluntary movement and are thus unable to communicate in any other way (Naito et al., 2007; Chaudhary et al., 2022). To date there has been limited investigation of clinical implementation of AAC-BCI technology. Several studies have described participants with disabilities using AAC-BCI systems independently in their own homes for extended periods (Sellers et al., 2010; Vansteensel et al., 2016; Wolpaw et al., 2018), but most have reported data from limited system use, often in laboratory environments (Chavarriaga et al., 2017).

There appears to be considerable variability in BCI performance across users, including for AAC-BCI systems. Previous research has found that a subset of users (estimated to be as many as 15–30% of all users) are unable to achieve BCI system control, a phenomenon sometimes referred to as “BCI illiteracy” (Blankertz et al., 2010). The concept of BCI illiteracy assumes the existence of normative information on performance in using these systems (Thompson, 2019), even though the field has yet to establish performance standards. Poor BCI system performance has been observed in users both with and without disabilities. It has been suggested that differences in brain activity, sensory skills, cognition, or age may have a negative effect on outcomes for those with disabilities (McCane et al., 2015; Geronimo et al., 2016). Many AAC-BCI studies include only participants without disabilities (Eddy et al., 2019), and it is unclear whether their results would generalize to individuals with SSPI, or whether there is a potential performance gap between these two groups of users.

Some researchers and end-users have taken issue with the term “BCI illiteracy,” given its association with difficulties in reading or writing and its implication that poor BCI system performance is related to shortcomings on the part of the user (“BCI inefficiency” has been proposed as an alternative, e.g., in Kübler et al., 2011). Thompson (2019) suggested that poor performance is a result not of user characteristics but rather of a mismatch between those characteristics and the demands of system use. An individual may have difficulty using a specific BCI system, and may demonstrate better performance when a different interface or control signal is trialed, or with modifications to display parameters. This is consistent with the concept of feature matching, widely used in the AAC and assistive technology fields to guide the selection of devices best suited to an individual's unique strengths, challenges, and preferences (Scherer and Craddock, 2002). A wide variety of AAC-BCI systems have been developed and investigated, including both implantable and non-invasive systems with a diverse array of interface types and control signals (Akcakaya et al., 2013; Rezeika et al., 2018; Pitt et al., 2019). Feature matching may be an appropriate framework for the selection of appropriate AAC-BCI systems for individuals with SSPI, but the existing body of research may not yet be sufficient to support such an approach (Pitt and Brumberg, 2018b; Brumberg et al., 2019). The BCI field has yet to agree on common standards for participant description or the reporting of system performance results (Allison and Neuper, 2010; Thompson et al., 2013; Eddy et al., 2019; Huggins et al., 2022), adding to the difficulty of interpreting and synthesizing evidence to guide clinical practice.

Given the rapid growth and interest in AAC-BCI research, it is critical that we evaluate the state of the science at regular intervals to determine if the field is advancing in the best directions for those whom the tools are meant to benefit. As this technology moves toward clinical implementation, stakeholders will need to know what types of systems have been tested and under what conditions, how well they work, and for whom. Researchers will need to ensure that study participants are representative of potential end-users, and that their methods and results are clearly reported. Although there have been multiple review chapters and articles on the current state of the science for AAC-BCI systems over the past 10 years, most have been non-systematic overviews of system types, algorithms, and applications, often describing results from studies that did not include participants with disabilities (Pasqualotto et al., 2012; Akcakaya et al., 2013; Alamdari et al., 2016; Chaudhary et al., 2016, 2021; Rezeika et al., 2018; Wang et al., 2019; Vansteensel and Jarosiewicz, 2020). Existing systematic reviews of AAC-BCI literature have focused on specific system types, such as non-visual interfaces (Riccio et al., 2012), or on the performance of specific end-user populations, such as people with ALS (Marchetti and Priftis, 2014) or cerebral palsy (Orlandi et al., 2021). We conducted a systematic review to evaluate the current state of AAC-BCI research involving participants with disabilities, with no restrictions on disability etiology or on AAC-BCI system type. The aims of this systematic review were to (1) describe study, system, and participant characteristics in the AAC-BCI literature, (2) summarize the communication task performance of participants with disabilities in AAC-BCI studies, and (3) explore any differences in AAC-BCI system performance for participants with and without disabilities. Table 1 summarizes the aims according to the PICOS framework (Akers et al., 2009).

**Table 1:** PICOS criteria for the systematic review.

P	Population	Individuals with speech and/or physical impairments
I	Intervention	AAC-BCI systems used for communication tasks
C	Comparison	Individuals without speech or physical impairments
O	Outcomes	Selection accuracy, selection speed/ITR, study and participant characteristics
S	Study designs	Any

## 2. Methods

This systematic review was conducted following the Preferred Reporting Items for Systematic Reviews and Meta-Analyses (PRISMA) framework. The protocol was registered with the international prospective register of systematic reviews (PROSPERO, CRD42018095345).

### 2.1. Identification of included studies

Studies included in the review met the following criteria: (1) included at least one adult participant with speech and/or physical impairments; (2) involved use of an AAC-BCI system for a communication task; (3) reported at least one outcome measure related to communication task performance (e.g., selection accuracy or ITR); (4) published in any year up to and including 2020; and (5) available in English. An AAC-BCI system was defined as a BCI system designed for the selection of characters, words, or symbols that could be used to communicate with another person. Some AAC-BCI studies involved a functional communication task, such as typing participant-generated messages or answering yes/no questions with known answers. Other studies directed participants to copy-spell words or phrases determined in advance by the investigators or select a designated response among two or more options (e.g., “yes” and “no” or a larger set of words or icons). Studies in which participants were instructed to attend to a stimulus or engage in mental imagery that was not associated with selection of characters, words, or symbols were excluded. There were no exclusion criteria related to design or methodological quality, as one goal of this review was to examine and describe the characteristics of BCI studies involving participants with disabilities. One study was excluded due to retraction of the article by the publisher.

Comprehensive searches were conducted by author GB in EbscoHost CINAHL, Ovid Medline, and Scopus in March, 2018, and updated by author BP in May, 2021. Studies were limited to adults and humans by keyword and subject terms, where appropriate, and date of publication was limited to 2020 or earlier. Search terms related to AAC were combined with terms related to BCI (e.g., “communication aids for disabled” OR “conversation” OR “typing” AND “brain-computer interface” OR “electroencephalography” OR “event-related potentials”). See Supplementary Materials for the exact terms and strategies used for each database. The reference lists of review articles were searched for additional relevant publications.

Records identified in the two searches were uploaded to Covidence, a web-based systematic review management platform (Covidence, Melbourne, Australia). After removing duplicate records, authors BP and BE screened record titles and abstracts and excluded those that did not meet inclusion criteria. Twenty-five percent of records were screened by both reviewers to allow assessment of inter-rater reliability, and the remaining 75% were split evenly between them by random assignment. Articles that appeared to meet criteria, or for which eligibility could not be determined based on title and abstract review, were evaluated further by both reviewers in the full-text review stage and discussed by both reviewers until consensus was reached. In the title and abstract review stage, the reviewers reached 97.4% agreement (Cohen's kappa 0.80). In the full-text review stage, they initially reached 95.2% agreement for inclusion (Cohen's kappa 0.87), resolved to 100% after consensus discussions.

### 2.2. Data extraction

Authors BP and BE reviewed each study that met inclusion criteria and extracted data related to study characteristics, AAC-BCI system characteristics and protocol description, participant characteristics and description, and communication task performance. Disagreements about subjective ratings, such as whether study or participant characteristics were adequately described, were resolved through discussion between BP and BE.

#### 2.2.1. Study characteristics

For each study, reviewers extracted data related to year of publication, experimental design, sampling strategy, reporting of inclusion and exclusion criteria, sample size, and collection and reporting of user experience feedback. We originally intended to assess study quality using a modified version of the Scale to Assess Scientific Quality of Investigations (SASQI) (Jeste et al., 2008), but found that the criteria did not apply to most of the identified studies due to their non-experimental designs.

#### 2.2.2. AAC-BCI system characteristics and protocol description

Reviewers recorded characteristics of the AAC-BCI system investigated in each study, including data acquisition method (e.g., electroencephalography [EEG] or implantable electrodes), interface type (e.g., visual, auditory, or tactile), and control signal (e.g., event-related potentials [ERP] or steady-state visual evoked potentials [SSVEP]). They also noted the communication task used for system performance evaluation. Finally, they reviewed whether studies clearly described each of 12 important AAC-BCI system and task protocol characteristics, such as control signal, method of signal processing, and communication task procedures. These characteristics were identified by authors BP, BE, and BO, and are listed in the Supplementary Materials.

#### 2.2.3. Participant characteristics and description

Reviewers extracted data on selected characteristics of participants with disabilities in each study, including age, diagnosis, duration of impairment or time since diagnosis, severity of speech and/or physical impairment, previous BCI experience, whether any participants were excluded from participation or analysis, and the reasons for exclusion, when applicable. For studies including participants without disabilities, the ages of those participants were extracted as well. Reviewers also determined whether important participant characteristics, such as diagnosis, cognitive skills, and sensory abilities, were adequately described. These characteristics were identified by authors BP, BE, and BO, and are listed in the Supplementary Materials. For characteristics that could be assessed using standardized instruments or other objective measures, reviewers noted whether a study reported such measurements or provided only narrative description.

#### 2.2.4. Communication task performance

Data extraction for communication task performance focused on descriptive statistics for selection accuracy (the number of correct letters, words, or other stimuli selected out of the total number of selections) and measures related to communication rate, such as information transfer rate (ITR), selections per minute, and correct selections per minute. Results of statistical comparisons of performance data for participants with and without disabilities were also extracted, where applicable. In cases where mean, standard deviation, or range were not reported, these were calculated based on the provided data whenever possible. When data were presented only visually, they were extracted from the visualization using an online tool (WebPlotDigitizer, Rohatgi, 2021). For studies in which participants without disabilities completed different tasks or conditions than those with disabilities, performance data were extracted only for the tasks or conditions completed by both groups. For studies involving BCI tasks not related to communication (e.g., navigating through a virtual environment or controlling a web browser), performance data were extracted only for the communication task(s). Several studies included a free-spelling task (in which participants chose their own words) in addition to a copy-spelling task (in which target words or phrases were determined in advance by the investigators and participants were instructed to copy the text). Only copy-spelling data were extracted from these studies, as the free-spelling tasks were typically not consistent across participants (i.e., the typed messages differed in length or complexity), and most reports of free-spelling results did not include measures such as accuracy or ITR. Free-spelling results were extracted from two studies that did not include a copy-spelling task, since those were the only performance results available.

To aid in summarizing the efficacy of AAC-BCI systems for people with disabilities, reviewers noted whether the mean or median accuracy reported in each study was 70% or higher, and the number of individual participants in each study who achieved 70% accuracy. The criterion of 70% accuracy as an indicator of successful communication task performance was first suggested by Kübler et al. (2001) and is now widely used in the BCI field.

### 2.3. Data management and reporting

Data were entered into a REDCap instrument (Harris et al., 2009, 2019) and exported into Excel spreadsheets. Data cleaning, exploration, and visualization, and calculation of descriptive statistics, were conducted in Tableau Prep Builder and Tableau Desktop (Tableau Software, Seattle, WA). Selected study characteristics, participant characteristics, and performance results were summarized in two tables: the first included all studies and focused on participants with disabilities, and the second presented data for participants with and without disabilities, extracted from studies that included both groups. In each table, studies were grouped by data acquisition method, interface type, and control signal. Some participant description terminology and performance measures were standardized for more consistent reporting of results and to facilitate data synthesis and comparison across studies. For example, a participant whose “motor abilities are reduced to two small facial muscle movements and weak eye movements which are exhausting and unreliable for extensive communication over longer time periods” (Hinterberger et al., 2003) was described as having incomplete LIS. ITR reported as bits per second was converted to bits per minute, and typing rate reported as seconds per character was converted to characters per minute. For these performance measures, both the original reported results and the standardized results were included in the study summary table. Descriptive statistics that could not be calculated based on the provided data were omitted from the study summary table.

## 3. Results

### 3.1. Included studies

**Figure 1:**
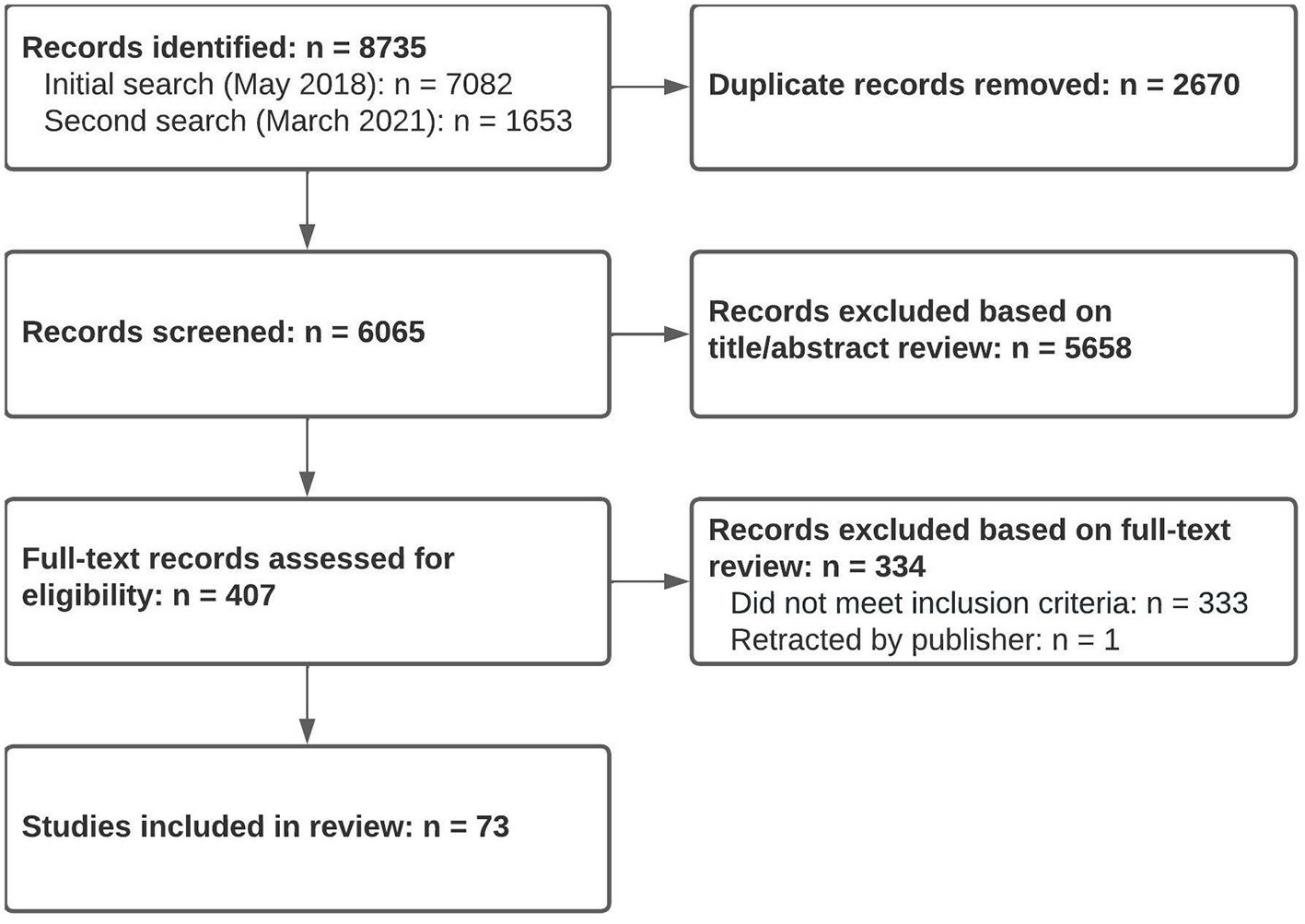
Study flow diagram.

A total of 6,065 studies were screened for eligibility, and 5,658 were excluded based on title or abstract review. An additional 334 were excluded after full-text review, leaving 73 studies which met eligibility criteria (see flow diagram in Figure 1).

### 3.2. Study characteristics

**Figure 2:**
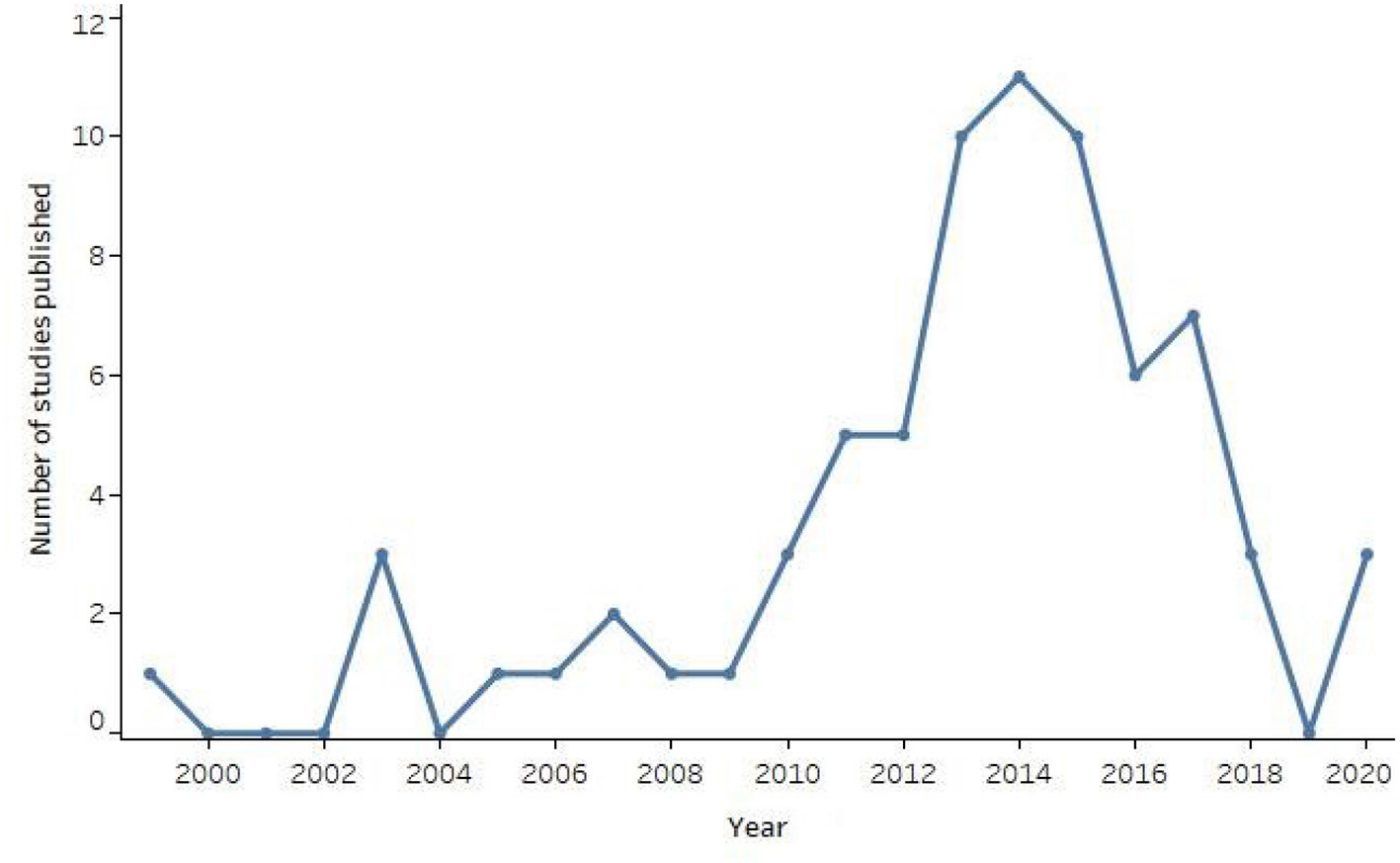
Number of included AAC-BCI studies published per year, 1999–2020.

**Figure 3:**
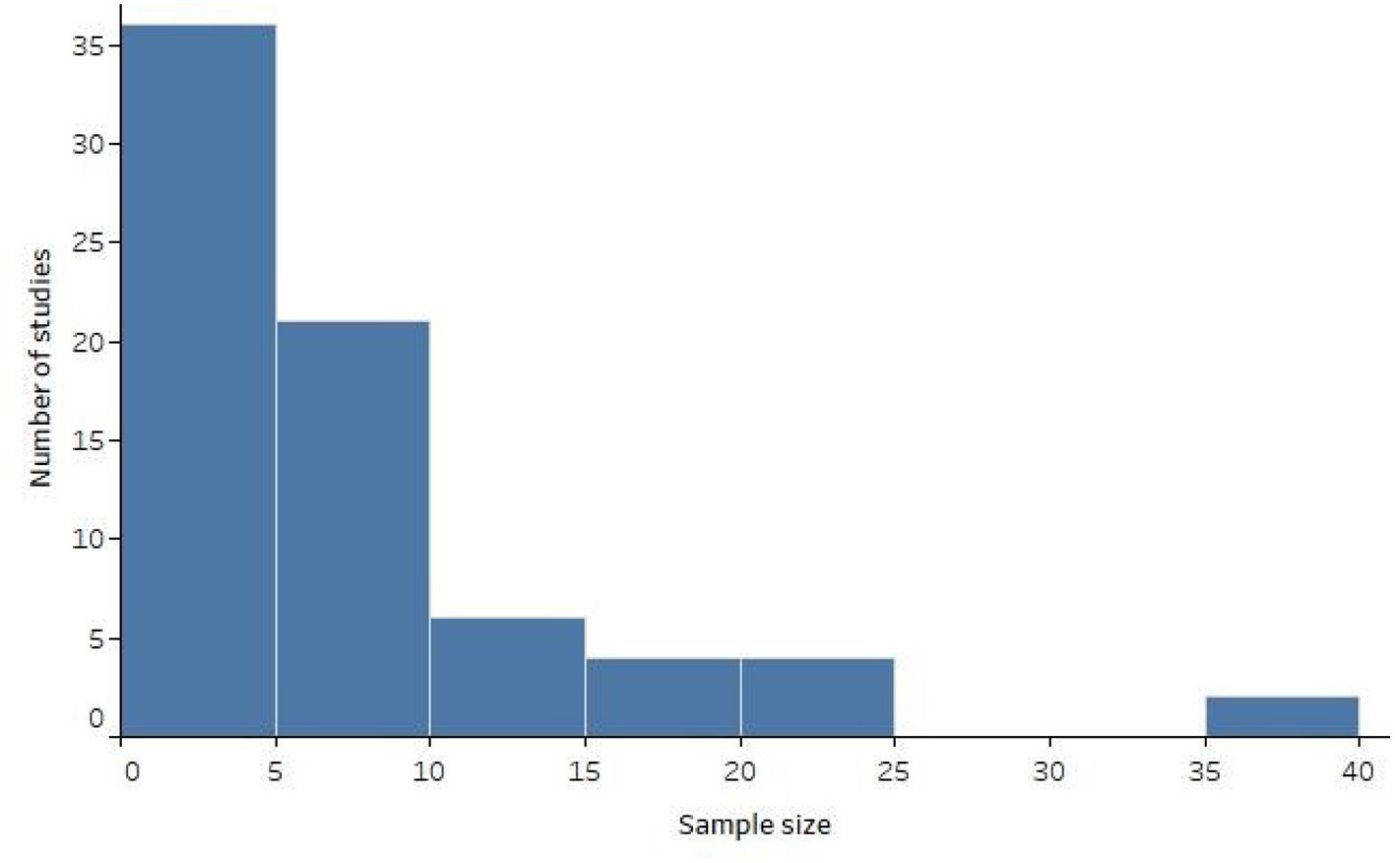
Distribution of study sample sizes for participants with disabilities.

Study characteristics and communication task results are summarized in Supplementary Table 1. The oldest study was from 1999, and the number of studies per year peaked in 2014 (see Figure 2). Most studies used non-experimental designs (*n* = 43, 58.9%), such as case studies or feasibility studies. Nine studies (12.3%) included a within-subjects or between-groups design for participants without disabilities, accompanied by a non-experimental design conducted with one or more participants with disabilities. One study (1.4%) reported use of consecutive sampling; the remaining studies either used convenience sampling or provided no information about sampling strategy. Inclusion and exclusion criteria were clearly described in 12 studies (16.4%). The distribution of study sample sizes for participants with disabilities is displayed in Figure 3. Sample sizes ranged from 1 to 40, with a median of 6 (interquartile range 1-10). Nineteen studies (26.0%) included only one participant with disabilities, and 36 studies (49.3%) included five or fewer.

Forty-one studies (56.2%) did not report any user experience feedback, and one (1.4%) reported feedback from participants without disabilities but not from those with disabilities. Among the 31 studies (42.5%) that reported feedback from individuals with disabilities, 22 used questionnaires with multiple choice, Likert-type, or visual analog scale formats, and 18 solicited narrative feedback with open-ended questions (nine used both approaches). One study reported participants' opinions about the BCI system but did not specify how those opinions were solicited. In some cases, participants with disabilities were unable to provide feedback due to the lack of a reliable non-BCI communication channel (e.g., participants with total LIS or disorders of consciousness, Naito et al., 2007; Lulé et al., 2013; Gallegos-Ayala et al., 2014; Guger et al., 2017). At least 19 studies that reported user experience feedback included participants with severe disabilities (e.g., participants with incomplete or classic LIS, or very low scores on the ALS Functional Rating Scale-Revised [ALSFRS-R], Cedarbaum et al., 1999).

### 3.3. AAC-BCI system characteristics and protocol description

Most AAC-BCI systems used EEG data (*n* = 66, 90.4%), often with ERPs as a control signal (*n* = 55, 75.3%). Three studies (4.1%) used implantable BCI systems, and four (5.5%) used functional near-infrared spectroscopy (fNIRS). AAC-BCI systems with visual interfaces were used in 56 studies (76.7%), most commonly variations on the classic P300 matrix speller (Farwell and Donchin, 1988), which appeared in 38 studies (52.1%) [The term “P300” as used in BCI literature includes both the N200 and P300 ERPs which both contribute to classifier performance (Patel and Azzam, 2005; Enriquez-Geppert et al., 2010), but interfaces using these ERPs have become commonly known as “P300-based” BCIs.]. Participants with disabilities tested auditory interfaces in ten studies (13.7%), tactile interfaces in four (5.5%), and audiovisual interfaces in two (2.7%). Some auditory or tactile interfaces used visual aids, such as a matrix of characters with rows and columns assigned to specific auditory or tactile stimuli. Six studies (8.2%) involved EEG- or fNIRS-based AAC-BCI systems without a typical user interface; participants in these studies were given instructions to produce two different mental states for response selection.

System performance was evaluated with a variety of communication tasks. Copy-spelling tasks were used in 49 studies (67.1%), with targets including words, phrases, sentences, icon sequences, and numeric or non-word character strings. Other tasks involved yes/no questions or selection of designated binary-choice response options (*n* = 14, 19.2%), selection of individual characters or icons (*n* = 9, 12.3%), free-spelling (used as the sole communication task for *n* = 2, 2.7%, and as an additional task for *n* = 6, 8.2%), or multiple-choice questions or response selection (*n* = 3, 4.1%).

**Figure 4:**
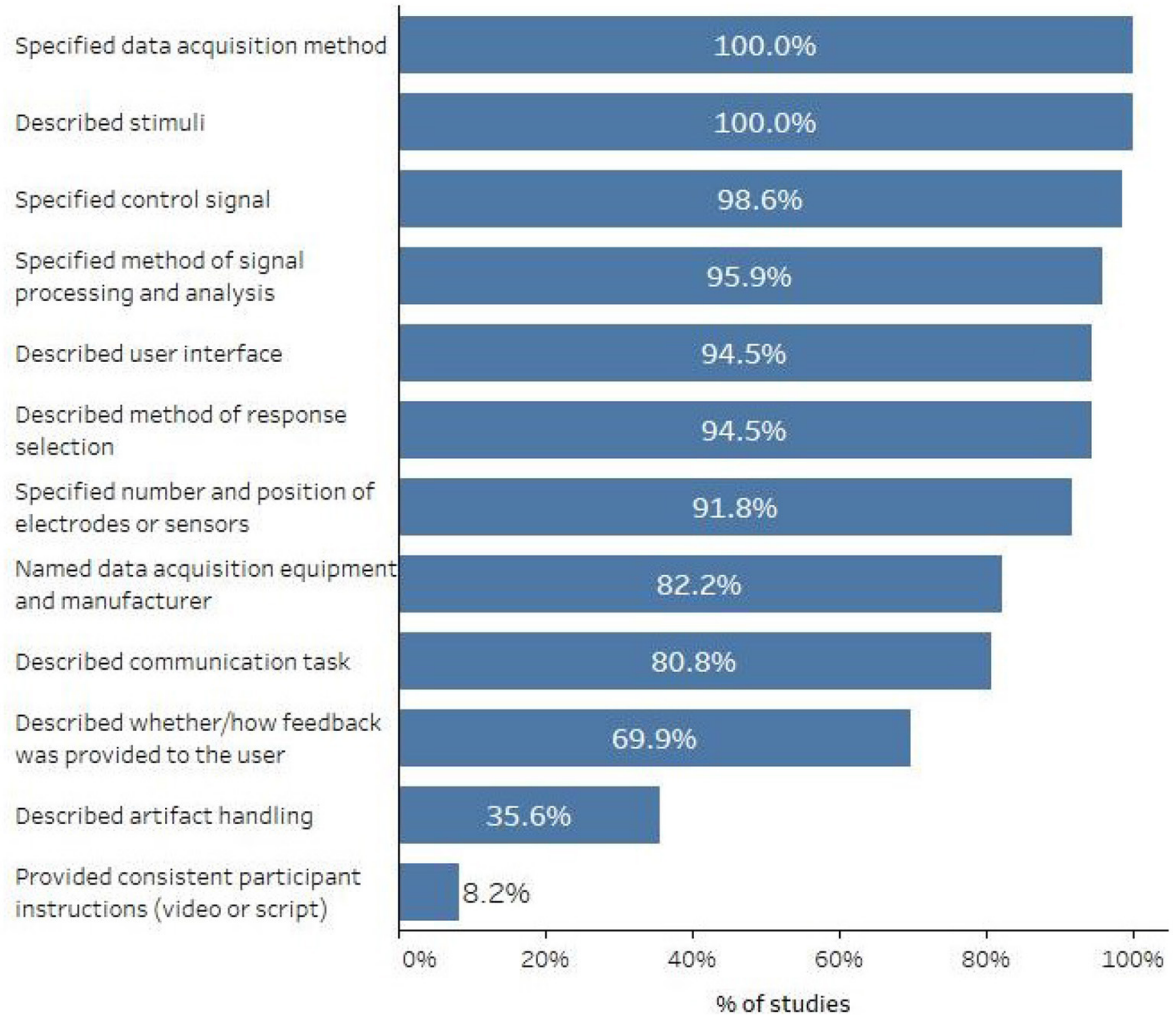
Percent of studies that described various system and protocol characteristics.

Figure 4 displays the percentage of studies that reported various AAC-BCI system or protocol characteristics. All studies specified the data acquisition method and the stimuli that elicited the brain response, and most described other system characteristics such as the control signal, methods of signal processing and classification, and user interface. Methods of handling artifacts (or a statement that no artifact handling methods were used) were included in 26 studies (35.6%). Only 6 studies (8.2%) mentioned that system or task instructions were provided to participants using a video or script, which would ensure procedural fidelity and consistent instructions across participants, or for the same participant over time.

### 3.4. Participant characteristics and description

Twenty-four studies provided information about whether any participants with disabilities withdrew or were excluded from participation or data analysis. Four indicated that no participants with disabilities had withdrawn or been excluded. A majority of studies (*n* = 49, 67.1%) provided no information about participant withdrawal or exclusion. Of the remaining 20 studies, 10 reported withdrawal of at least one participant due to scheduling or logistical challenges, illness, death, or other reasons. Reasons for participant exclusion included: participant characteristics such as fatigue, cognitive impairment, or muscle artifacts (*n* = 11); not meeting a specified BCI performance threshold (*n* = 8); poor signal quality or other technical difficulties (*n* = 5); and inability to provide informed consent (*n* = 1).

**Figure 5:**
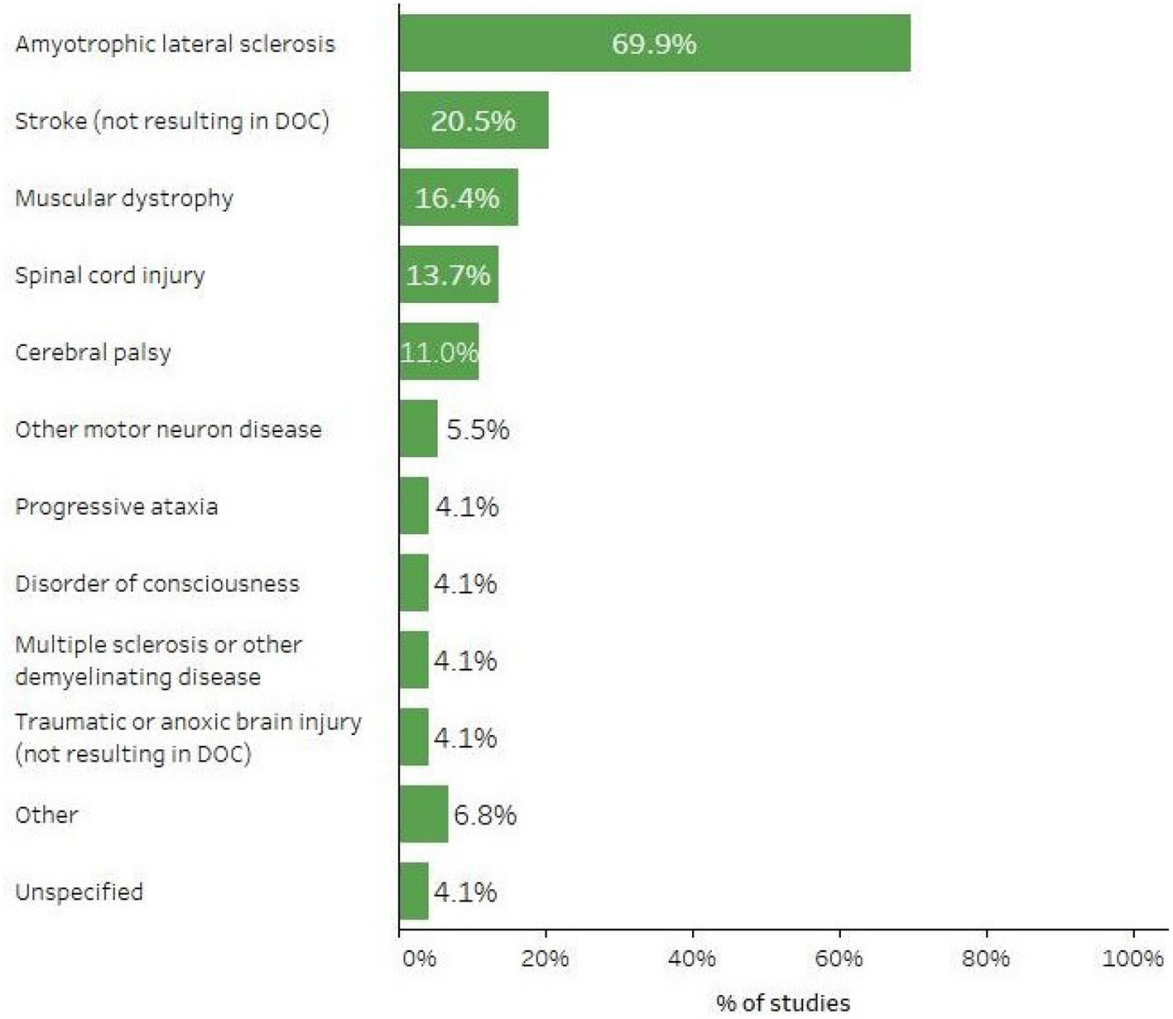
Percent of studies including participants with specific diagnoses or conditions.

Forty-eight studies (65.8%) involved participants with disabilities arising from a single diagnosis, while the others reported a variety of participant diagnoses. Overall, ALS was the most frequently-reported diagnosis for AAC-BCI study participants; individuals with ALS were included in 51 studies (69.9%). See Figure 5 for a summary of the percentage of studies including participants with various diagnoses. Participants with disabilities ranged from 17 to 90 years of age, but a majority of studies (*n* = 60, 82.2%) reported mean or median ages, or the age of a single participant in a case study, between 40 and 70 years.

Some studies used LIS or its categories (incomplete, classic, and total) to indicate the severity of participants' speech and physical impairments, while others listed conditions such as tetraplegia or dysarthria, or described participants' ability to use specific body parts such as the eyes or extremities. The most commonly used standardized measure of physical disability was the ALSFRS-R (Cedarbaum et al., 1999), reported in 31 studies (43.8%). One study used the original ALSFRS (ALS CNTF Treatment Study (ACTS) Phase I-II Study Group et al., 1996). All studies using the ALSFRS or ALSFRS-R reported total scores only, with no information about domain subscores. Other standardized instruments and scales included the American Spinal Injury Association Impairment Scale (*n* = 4, 5.5% Roberts et al., 2017), the Gross Motor Function Classification System (*n* = 1, 1.4% Palisano et al., 1997), and the Coma Recovery Scale-Revised (*n* = 1, 1.4%, Kalmar and Giacino, 2005).

**Figure 6:**
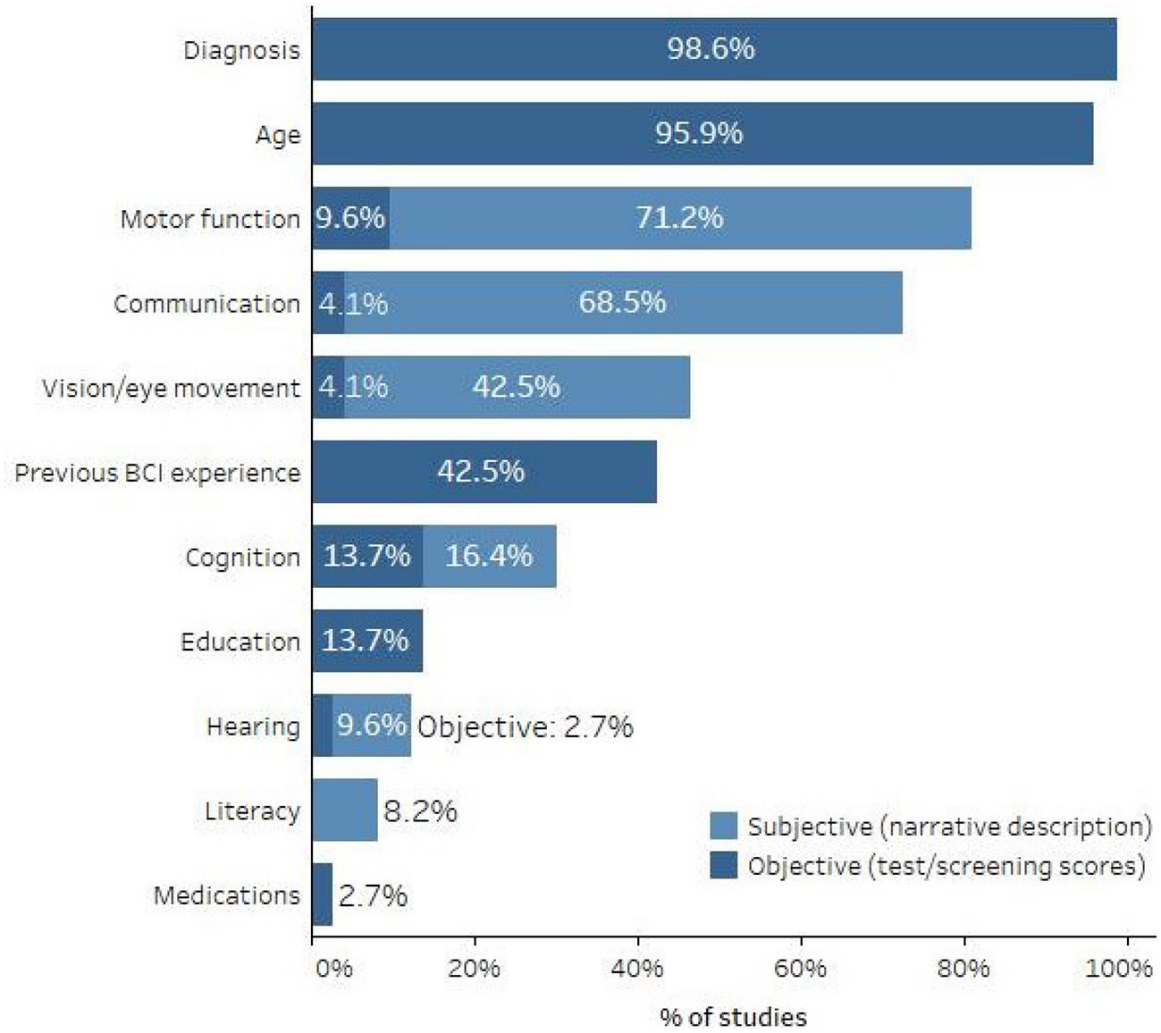
Percent of studies that described various participant characteristics.

There was substantial variability in the duration and severity of disability among study participants, as well as in the level of detail with which they were described. While most studies specified the diagnoses and ages of their participants (*n* = 72 and *n* = 70, respectively), description of additional characteristics, such as previous BCI experience, cognitive status, physical and sensory abilities, and current communication methods, was less common. Figure 6 shows the percentage of studies that reported these and other participant characteristics, and for some measures, whether they were reported in the form of standardized test or screening scores or narrative descriptions. Because individuals with similar ALSFRS-R total scores can have very different clinical presentations (e.g., one could have strong bulbar function but poor fine motor function while another could have the opposite presentation but the same ALSFRS-R score), total scores were not considered an appropriate test score for describing motor function unless the reported score was 0.

### 3.5. Communication task performance measures and results

Most studies (*n* = 68, 93.2%) reported on the accuracy of participants' communication task performance. However, accuracy was not measured or reported consistently across studies, which makes it difficult to compare performance even for similar AAC-BCI systems. Communication task instructions (e.g., whether a participant should correct an error or ignore it and move on to the next target, which might change the calculated accuracy) and system configurations (e.g., higher or lower numbers of stimulus presentations in an ERP-based speller, which might make it easier or harder to make accurate selections) varied greatly across research groups. The participants for whom accuracy was reported also varied across studies. Eight studies (11.0%) reported accuracy only for participants who reached a specified performance threshold indicating successful system control, while others reported results from all participants who attempted a communication task, regardless of performance. In studies involving multiple sessions with the same communication task, some reported separate accuracy results for each session, while others reported average accuracy across sections, or only the maximum accuracy achieved by each participant.

Forty-five studies (61.6%) reported a mean or median selection accuracy of at least 70% on one or more communication task conditions for participants with disabilities. However, eight of these studies reported excluding one or more individuals with disabilities from study participation or data analysis due to poor system performance, and another 30 did not specify whether any participants had been excluded for this reason. In 23 studies (31.5%), all participants reached the 70% accuracy criterion in at least one tested condition. Eleven studies (15.1%) had no participants who reached the criterion, and 26 studies (35.6%) reported mixed results. Thirteen studies did not report accuracy results at the individual participant level, or at all.

Some studies that compared performance across more than one condition found that more participants were successful under one condition than another. For example, Townsend et al. (2010) found that three of three participants achieved an accuracy of 70% or better with a checkerboard flashing paradigm on a matrix speller, but none were successful with a row-column flashing paradigm. Combaz et al. (2013) compared an ERP-based system to an SSVEP-based system, with all seven participants reaching the 70% accuracy criterion using the SSVEP-based system and only three of seven doing so with the ERP-based system. Severens et al. (2014) compared a tactile speller and a visual speller; four of five participants achieved 70% accuracy or better with the visual speller, but only one did so with the tactile speller. Other user or system characteristics that appeared to demonstrate a possible correlation with system performance included visual impairment (McCane et al., 2014), electrode type (Clements et al., 2016), error correction (Riccio et al., 2015), and number of sequences displayed (Kaufmann et al., 2013b).

Approximately half of studies (*n* = 37, 50.7%) reported at least one performance measure other than accuracy. Measures of ITR, bit rate, bandwidth, or practical bit rate were most common, appearing in 25 studies (34.2%). Researchers calculated ITR or bit rate in various ways, including Wolpaw's method (Wolpaw et al., 2002) and Nykopp's method (Nykopp, 2001). Other measures included selections (characters, symbols, or keystrokes) per minute (*n* = 10, 13.7%), correct characters per minute (*n* = 3, 4.1%), seconds per character (*n* = 3, 4.1%; converted to characters per minute in Supplementary Tables 1, 2), seconds per correct selection (*n* = 1, 1.4%; converted to correct selections per minute in Supplementary Tables 1, 2), and seconds to spell a common (unspecified) sentence (*n* = 1, 1.4%). Some studies included more than one of these measures. Mean values for ITR ranged from 0.17 to 144 bits/min, and mean values for selections per minute or correct characters per minute ranged from 1.3 to 28.1.

### 3.6. Comparisons to participants without disabilities

Twenty-nine studies (39.7%) reported results from both participants with disabilities and control participants without disabilities, and are summarized in Supplementary Table 2 (see Supplementary Table 1 for additional study details). There was a wide range of reported tasks, system configurations, and/or study designs, and participants with and without disabilities did not always complete the same tasks or use the same system configurations. Of the 27 studies that reported mean accuracy for participants both with and without disabilities, a mean of at least 70% accuracy on one or more communication tasks was reported for control participants in 26 studies and for participants with disabilities in 18 studies.

Only eight studies reported a statistical between-groups comparison. Four of those analyses found no significant performance differences between participants with and without disabilities (Sellers et al., 2006; Ikegami et al., 2011; Oken et al., 2014; McCane et al., 2015), though a comparison of the highest difficulty level completed on a multi-level typing task approached significance for one study (Oken et al., 2014). One study found significantly higher accuracy and ITR for participants without disabilities (Nam et al., 2012), while two others found significant performance differences for a classic P300 matrix speller design but not for modified versions [flashing with famous or personally-known faces in Kaufmann et al. (2013b) and two-step selection in Ikegami et al. (2014)]. Finally, one study found a significant difference when comparing participants without disabilities to people with ALS and cognitive impairment, but not when comparing them to people with ALS and intact cognition (Geronimo et al., 2016).

## 4. Discussion

This systematic review identified 73 studies, published from 1999 to 2020, that reported AAC-BCI system performance results for adult participants with disabilities. The frequency of publication for these studies peaked between 2013 and 2015, with 10 to 11 studies per year, and has decreased in the more recent past with only three studies published each year in 2018 and 2020, and none in 2019. Although certain system types (e.g., the P300 matrix speller) and participant categories (e.g., individuals with ALS) were more well-represented than others, these studies reflect a wide array of approaches to AAC-BCI system design and featured participants with varying levels of disability and underlying diagnoses.

### 4.1. Study characteristics

Many studies used non-experimental designs, often with small sample sizes. While this is appropriate for feasibility testing and preliminary investigations, more rigorous experimental designs will be needed in the future to support clinical implementation of AAC-BCI systems. There are several potential challenges that may affect the inclusion of participants with disabilities in AAC-BCI research. It may be difficult to recruit large numbers of participants with similar conditions, especially for those with rare disorders. In addition, participants with severe disabilities may be unwilling or unable to travel to a laboratory for data collection. Home-based, longitudinal studies such as those conducted by the Wadsworth Center, with internet-based technical support and data collection procedures, may serve as a model for conducting AAC-BCI research with larger sample sizes (Wolpaw et al., 2018). Single-case experimental research designs in which participants serve as their own controls, like the alternating-treatments design used by Peters et al. (2020), are a good option for future AAC-BCI studies. Such designs are common in the behavioral sciences, including communication sciences, and allow researchers to draw scientifically valid inferences about the effects of an intervention from the performance of a small number of participants (Horner et al., 2005; Krasny-Pacini and Evans, 2018; Kazdin, 2019). The flexible nature of single-case experimental research designs permits investigators to modify an intervention mid-experiment to address individual participants' needs and preferences, fitting well within the iterative user-centered design approach often recommended for BCI research and development (Chavarriaga et al., 2017; Choi et al., 2017; Garro and McKinney, 2020; Kubler, 2020; Alonso-Valerdi and Mercado-Garcia, 2021; Branco et al., 2021; Pitt and Brumberg, 2021; Han et al., 2022).

Fewer than half of the studies summarized in this review reported the collection of user experience feedback from participants with disabilities. To ensure that AAC-BCI systems will meet users' needs and preferences in future clinical implementation, individuals with disabilities must not only be included as participants in performance evaluation studies, they must provide input into the development and refinement of the systems themselves. In user-centered design, researchers must understand users' needs and preferences, consider those needs and preferences in the design process, and evaluate and iterate their designs in response to user feedback (Kübler et al., 2014). Several studies solicited user experience feedback from participants with disabilities using questionnaires, open-ended questions, or a combination of methods. See Peters et al. (2016) for suggestions on AAC-based approaches to obtaining feedback from individuals with severe speech and physical impairments that may preclude the use of standard methods.

Most AAC-BCI studies included in this review either used convenience sampling or did not describe their sampling methods. Inclusion and exclusion criteria were often missing or vague. Of the studies that specified inclusion or exclusion criteria based on BCI performance, some reported the number of potential participants who did not achieve the required performance target. However, other studies gave no indication of whether any potential participants had been excluded due to poor initial BCI performance. Thus, study results may not be representative of performance for a randomly-selected group of people with disabilities with similar characteristics. BCI study reports should include a clear description of inclusion or exclusion criteria, indicate the total number of potential participants who were screened for inclusion, and state the reasons for exclusion of any screened individuals. We recommend the CONSORT flow diagram format (without the randomization arms, as appropriate) as a useful model for organizing and reporting this information (Moher et al., 2001; CONSORT Group, n.d.). While the exclusion of people not meeting a given performance criterion may be appropriate for many studies, such as those exploring the effects of system modifications, it should be reported nonetheless to avoid misrepresentation of system performance. Transparency around who is included in BCI studies, who is excluded, and why will present a more realistic portrait of the overall effectiveness of BCI for users with disabilities, and highlight areas for future research and development to address the needs of people not well served by existing systems.

### 4.2. AAC-BCI system characteristics

Most AAC-BCI studies involving participants with disabilities focused on non-invasive, EEG-based systems, with ERPs as the most common control signal. Over half of the studies in this review investigated variations on the P300 matrix speller, consistent with previous reviews showing frequent use of this paradigm (Rezeika et al., 2018). Most AAC-BCI systems used visual interfaces, with limited exploration of alternatives such as auditory or tactile interfaces that might benefit users with visual or ocular motility impairments. Such impairments are common among individuals with ALS, LIS, or other conditions causing SSPI (Fried-Oken et al., 2020), so non-visual interfaces will be an important area for future research. A majority of the visual and auditory interfaces were text-based, with communication tasks requiring participants to read and/or spell words. Further investigation of paradigms involving symbols, icons, or auditorily-presented words, such as those described in several included studies (Neshige et al., 2007; Lulé et al., 2013; Marchetti et al., 2013; Silvoni et al., 2013; Hill et al., 2014; Scherer et al., 2015), will provide options for AAC-BCI users who have difficulty with text due to their cognitive or literacy skills.

### 4.3. Participant characteristics

Participant characteristics that might affect AAC-BCI system performance and user experience were seldom clearly described. For example, fewer than half of studies reported vision screening results or descriptions of visual acuity or ocular motility, even though visual AAC-BCI interfaces were frequently used. One study identified a correlation between visual impairment and poor copy-spelling performance with a P300 matrix speller (McCane et al., 2014), highlighting the importance of considering such characteristics in study design, and of reporting them clearly. Certain medications, including many commonly used by individuals with severe speech and physical impairments, have been shown to alter EEG (Blume, 2006; Polich and Criado, 2006), but only two studies made any mention of participant medications. Cognitive function and literacy were also rarely described, despite their relevance to the use of complex, and often text-based, AAC-BCI systems. For example, people with ALS, who were the most common participants in AAC-BCI studies, often experience cognitive changes ranging from mild impairment to frontotemporal dementia (Beeldman et al., 2016), which could affect their performance on communication tasks. In addition to communication task performance, cognitive, sensory, language, and literacy skills may affect whether participants can hear, see, and understand instructions for system operation and completion of experimental tasks. To ensure study replicability, and to provide an evidence base for feature matching and selection of a system appropriate to an individual's strengths and challenges, AAC-BCI researchers must clearly describe participant characteristics that are relevant to system use. Screening tools such as those proposed by Fried-Oken et al. (2015) and Pitt and Brumberg (2018a) may support the collection and reporting of these data. International efforts to standardize description of participants and their environments are under way (Huggins et al., 2022).

Disability type and severity were also unclear in many studies, a potentially problematic omission given the apparent relationship between severe disability (particularly total LIS) and poor BCI performance. Many BCI studies, including many of those summarized in this review, report total ALSFRS-R scores as a measure of participants' physical functioning. Although total ALSFRS-R scores can roughly indicate an individual's overall level of impairment, especially at the high and low ends of the scoring range, reporting the four ALSFRS-R component domain scores (bulbar, fine motor, gross motor, and respiratory) would be a more detailed and specific means of describing the physical disabilities experienced by BCI study participants (Franchignoni et al., 2013; Bacci et al., 2016). Given that individuals with LIS are often specified as the target users of AAC-BCI systems, it would be helpful to specify LIS categories (incomplete, classic, and total, Bauer et al., 1979; Smith and Delargy, 2005) in participant descriptions. This would allow readers to easily identify whether study participants presented with a level of disability consistent with the target user population.

### 4.4. Communication task performance

Communication task performance in AAC-BCI studies for participants with disabilities was highly variable both within and across studies. Some studies reported that all of their participants achieved 70% accuracy or better on a communication task, while others had no participants demonstrating successful task completion. Still others reported mixed results, often with wide ranges in communication task accuracy across participants. Many studies did not indicate whether any individuals with disabilities had been excluded from participation or data analysis due to poor system performance, making it difficult to interpret results indicating high accuracy on a performance task. Performance on other measures such as ITR and selections per minute also differed considerably within and across studies. Few studies reported statistical analysis comparing AAC-BCI system performance for participants with and without disabilities, and results for those that did were mixed. To date, there is insufficient evidence to either support or refute the existence of a performance gap. Researchers and other stakeholders should exercise caution in interpreting the results of studies in which participants without disabilities test technology intended for people with disabilities.

The variable results reported in these studies highlight the fact that there is no “one-size-fits-all” AAC-BCI system for individuals with severe speech and physical impairments. This review demonstrates that a diverse array of AAC-BCI systems have been tested by participants with disabilities, including both non-invasive and implantable systems as well as various interface types and configurations. Several studies that compared two conditions [e.g., visual instead of tactile (Severens et al., 2014), SSVEP instead of P300 (Combaz et al., 2013), or checkerboard flashing instead of row-column flashing (Townsend et al., 2010)] found that participants could be more successful with some AAC-BCI systems than others, indicating that “BCI illiteracy” may be a consequence of system design rather than participant characteristics or abilities. Instead of declaring some individuals to be incapable of BCI use, we must change and improve the technology to adjust to user needs and preferences. As Thompson (2019, p. 1231) put it in her critique of BCI illiteracy, “A BCI that doesn't work for its users doesn't work.” Development, evaluation, and refinement of new AAC-BCI systems and interfaces, and ensuring that they are customizable for individual users, will provide the variety of options needed to support feature matching in clinical implementation.

Rehabilitation scientists and clinicians, such as speech-language pathologists and occupational therapists, play a vital role in an interdisciplinary BCI research team. Approaching AAC-BCI systems from a clinical perspective, they bring an understanding of functional limitations, and of accommodations that can reduce challenges often faced by people with disabilities. They can support the implementation of many of the recommendations outlined here, including participant screening and description, communication with participants to facilitate user-centered design and collection of user experience feedback, and advocating on behalf of users with disabilities throughout the research and development process. As the field moves closer to clinical implementation of AAC-BCI systems, incorporation of the clinical perspective will ensure that the technology moves beyond impressive feats of engineering to become relevant and useful for individuals with disabilities.

### 4.5. Limitations

An important limitation of this systematic review is the lack of ratings of study design and evidence quality. Although such ratings had been planned and were outlined in the original PROSPERO protocol, many of the questions in the modified SASQI tool were inapplicable to the non-experimental designs found in the bulk of these studies. We decided to focus instead on the reporting of specific study elements such as participant and protocol description and inclusion and exclusion criteria. Readers should be mindful of the unknown quality of the evidence when reviewing these results. In addition, the 70% accuracy criterion as a measure of “successful” AAC-BCI system performance, while widely used in the literature, may not adequately capture the performance of systems with different chance levels (Billinger et al., 2012), and should be interpreted with caution. The PROSPERO protocol also proposed a meta-analysis of comparisons of AAC-BCI system performance for participants with and without disabilities, but this was not possible due to the small number of studies reporting statistical comparisons and to considerable differences in the systems and communication tasks used in those studies. Such differences, as well as inconsistencies in participant description and outcome measure reporting, limited our overall ability to synthesize the results of these studies.

## 5. Conclusion and recommendations

This systematic review provides an overview of AAC-BCI research involving adult participants with disabilities reported through 2020. It reveals the state of study design and reporting in this growing interdisciplinary field, characterized by small sample sizes, frequent use of non-experimental designs, and incomplete and inconsistent description of study and participant characteristics. To support the development of effective AAC-BCI systems and improve the evidence base for future clinical implementation, a number of recommendations emerge from these data. First, more rigorous experimental designs must be implemented, and efforts must be made to include larger samples of participants with disabilities when appropriate for the study design. An alternative to larger sample sizes would be the use of single-case experimental research designs (also known as N-of-1 designs), a scientifically rigorous means of exploring system performance with small sample sizes. Peer-reviewed articles should provide clear descriptions of inclusion and exclusion criteria, as well as reports about whether any participants were excluded from participation or analysis, and why (ideally presented in a flow diagram as recommended by the CONSORT Group; Moher et al., 2001; CONSORT Group, n.d.). There must be clear descriptions of participant characteristics that may affect system performance or the ability to engage in study tasks, and of system and protocol characteristics. Whenever possible, research and development should be conducted by interdisciplinary teams, including individuals with disabilities as well as clinicians with experience working with people with disabilities. A user-centered design approach, with use of AAC-based methods for eliciting user experience feedback when appropriate, should guide system design and iterative development. Finally, the field must continue its collaborative efforts to standardize the reporting of participant characteristics and performance outcome measures, such as those begun during workshops at the international BCI Meeting series (Huggins et al., 2022).

The AAC-BCI field is developing and evaluating new clinical technologies at a rapid pace. Research considerations that were unimaginable a decade ago are now essential as we move toward developing an evidence base to support clinical implementation. Alongside the development of new technologies, we must remain mindful of the ultimate goals of our work, and take time for self-evaluation and self-criticism of our methodologies and practices. Through rigorous research and dissemination, interdisciplinary collaboration, and, crucially, the involvement of potential end-users, we can lay the groundwork for successful clinical implementation of AAC-BCI systems, bringing innovative new communication options to individuals with severe speech and physical impairments.

## Data availability statement

The raw data supporting the conclusions of this article will be made available by the authors, without undue reservation.

## Author contributions

BP, BE, GB, BO, and MF-O contributed to the conception and design of this systematic review. GB conducted the initial database search, which was later updated by BP. BP and BE reviewed articles for inclusion and extracted data. All authors contributed to manuscript writing, revision, and approved the submitted version.

## Funding

This work was supported by the National Institutes of Health under Grant #DC009834.

## Conflict of interest

The authors declare that the research was conducted in the absence of any commercial or financial relationships that could be construed as a potential conflict of interest.

## Publisher's note

All claims expressed in this article are solely those of the authors and do not necessarily represent those of their affiliated organizations, or those of the publisher, the editors and the reviewers. Any product that may be evaluated in this article, or claim that may be made by its manufacturer, is not guaranteed or endorsed by the publisher.
