# Supplementary material for: A systematic review of research on augmentative and alternative communication brain-computer interface systems for individuals with disabilities
Source: Front Hum Neurosci. 2022 Jul 27;16:952380. doi: 10.3389/fnhum.2022.952380 (PMC9374067; doi:10.3389/fnhum.2022.952380)
Supplement: Supplementary file 1 [file Table_1.docx]

**Supplementary table 1.** Summary of key study, system, and participant characteristics, and communication task performance, for all studies included in the systematic review. All sample sizes, participant characteristics, and communication task performance data in this table are for participants with disabilities. See supplementary table 2 for results from participants without disabilities. “ERP” refers to P300 and N200 signals. Studies are organized first by interface type and then in alphabetical order. For studies reporting accuracy for a smaller sample than that indicated in the sample size column (i.e., due to participant exclusion), the sample size for accuracy calculation is indicated. N/R=not reported. M=mean. SD=standard deviation. R=range. SEM=standard error of the mean. Med=median. IQR=interquartile range. Dx=Diagnosis. n=sample size. Ex=excluded. Wi=withdrew. Standard medical abbreviations were provided for medical diagnoses. ALS=amyotrophic lateral sclerosis. OPCA=olivopontocerebellar atrophy. CP=cerebral palsy. MD=muscular dystrophy. SCI=spinal cord injury. CVA=cerebrovascular accident or stroke. SMA=spinal muscular atrophy. MS=multiple sclerosis. SBMA=spinobulbar muscular atrophy. LSM=lipid storage myopathy. PLS=primary lateral sclerosis. FA=Fredrich’s ataxia. DOC=disorders of consciousness. S/p=status post. TBI=traumatic brain injury.

| **Study** | **Interface**  **description** | **Task**  **(target)** | **Conditions or sessions** | **Sample**  **size** | **Diagnosis** | **Age**  **(years)** | **Time**  **since dx** | **Level of impairment** | **n with prior BCI exp.** | **Excluded/ withdrew, reason** | **Accuracy results (%)** | **n with**  **≥70%**  **accuracy** | **Other**  **measures** |
| --- | --- | --- | --- | --- | --- | --- | --- | --- | --- | --- | --- | --- | --- |
| ***EEG, ERP, Visual*** | | | | | | | | | | | | | |
| Carabalona et al., 2012 | Matrix speller | Copy-spell (*word & icon*) | N/A | 9 | ALS (n=1), MND (n=1),  MS (n=5), FA (n=1), demyelina-ting disease of CNS (n=1) | M=50.3 SD=9.7  R=38-66 | M=11.1 SD=8.0  R=1-26 years | N/R | 0/9 | N/R | *Word*  Med=80  IQR=50-90 *Icon*  Med=5  IQR=33-67 | N/R | N/R |
| Caruso et al., 2013 | *Matrix speller & prototype keyboard* | Copy-spell (sentence) | N/A | 3 | N/R | M=63.3 | N/R | ALSFRS-R: M=28.0  R=9-38 | N/R | N/R | *Matrix*  M=98.3 *Prototype* M=96.3 | 3/3 | N/R |
| Clements et al., 2016 | Matrix speller | Copy-spell  (word) | 2 conditions:  *wet & dry electrodes* | 8 | ALS (n=7),  PLS (n=1) | N/R | N/R | ALSFRS-R:  M=25.4  SD=15.5  R=4-46 | N/R | Ex: vision loss or caregiver  availability (n=3) | *Wet*  M=87.9  R=67-100  *Dry*  M=50.9  R=3-90 | *Wet*  7/8  *Dry*  2/8 | Bit rate (bits/min):  *Wet*  M=32.5  *Dry*  M=8.9 |
| Cruz, Pires, & Nunes, 2018 | Lateral single character speller | Copy-spell (sentence) | 2 conditions:  *with & without error correction (EC)* | 1 | SCI | N/R | N/R | Tetraplegia | 0/1 | N/R | *With EC* M=79.6; *Without EC*  M=75.0 | 1/1 | ITR (bits/min):  *With EC*  M=9.5  *Without EC*  M=8.6 |
| Geronimo, Simmons, & Schiff, 2016 | Matrix speller | Copy-spell  (word) | N/A | 25 | ALS | Med=58  R=45.5-74 | Med=32  R=12-113 months | ALSFRS-R: Med=30  R=0-46 | N/R | Ex: dementia (n= N/R) | M=68  R=0-100 | 16/25 | N/R |

| **Study** | **Interface**  **description** | **Task**  **(target)** | **Conditions or sessions** | **Sample**  **size** | **Diagnosis** | **Age**  **(years)** | **Time**  **since dx** | **Level of impairment** | **n with prior BCI exp.** | **Excluded/ withdrew, reason** | **Accuracy results (%)** | **n with**  **≥70%**  **accuracy** | **Other**  **measures** |
| --- | --- | --- | --- | --- | --- | --- | --- | --- | --- | --- | --- | --- | --- |
| ***EEG, ERP, Visual (continued)*** | | | | | | | | | | | | | |
| Guy et al., 2018 | Matrix speller | Copy-spell  (word) | N/A | 20 | ALS | M=60.7 SD=13.9  R=37-90 | M=6.1 SD=4.1  R=1-15 years | ALSFRS-R:  M=25  SD=8.1  R=8-37 | N/R | Ex: did not achieve system control (n=N/R); Wi: fatigue (n=1) | M=97.5 SD=3.4  R=90-100 | 20/20 | N/R |
| Ikegami et al., 2011 | Matrix speller | Character selection | 2 conditions: *green-blue* & *white-gray flickering* | 10 | SCI | M=41.9 SD=7.6  R=26-53 | M=18.2 SD=8.0 R=5.5-29.2 years | ASIA: incomplete (n=5), complete (n=5; grades unspecified) | 0/10 | N/R | *Green-blue*  M=90.7  SEM=1.0  *White-gray*  M=88  SEM=1.2 | N/R | Bit rate  (bits/min):  *Green-blue*  M=10.2  *White-gray*  M=9.8 |
| Ikegami et al., 2014 | Matrix speller | Character  selection | 2 conditions: *row-column & two-step (region followed by individual characters) flickering* | 7 | ALS | M=64.1 SD=3.3  R=59-68 | M=6.1  SD=4.9  R=2.2-16.2 years | ALSFRS-R:  M=9.4  SD=13.5  R=0-38 | 0/7 | N/R | *Row-column*  M=23.8  SD=13.1  R=16.7-50  *Two-step* M=54.8  SD=31.5  R=16.7-100 | *Row-column*  0/7  *Two-step* 2/7 | N/R |
| Käthner, Kübler, & Halder, 2015 | Matrix speller | Copy-spell  (word) | 3 displays:  *32" TV;*  *22" monitor; virtual reality (VR) headset* | 1 | ALS | 80 | N/R | ALSFRS-R: 0;  Classic LIS | N/R | N/R | *TV display* M=33  *22" monitor*  M=50.3  R=18-67  *VR*  M=45.3  R=17-100 | 0/1 | N/R |
| Käthner et al., 2017 | Matrix speller | Copy-spell  (word) | 2 electrode types: *wet & dry* | 6 | ALS (n=1),  CVA (n=1),  CP (n=2),  SCI (n=1),  LSM (n=1) | M=47.3 SD=14.3 R=26-62 | N/R | Tetraparesis (n=3), tetraplegia (n=1),  hemiparesis (n=1),  LIS unspecified (n=1) | N/R | N/R | Maximal accuracy  n=3  M=76.7  R=60-100 | 2/6 | N/R |

| **Study** | **Interface**  **description** | **Task**  **(target)** | **Conditions or sessions** | **Sample**  **size** | **Diagnosis** | **Age**  **(years)** | **Time**  **since dx** | **Level of impairment** | **n with prior BCI exp.** | **Excluded/ withdrew, reason** | **Accuracy results (%)** | **n with**  **≥70%**  **accuracy** | **Other**  **measures** |
| --- | --- | --- | --- | --- | --- | --- | --- | --- | --- | --- | --- | --- | --- |
| ***EEG, ERP, Visual (continued)*** | | | | | | | | | | | | | |
| Kaufmann et al., 2013b | Matrix speller | Copy-spell  (word) | 3 conditions:  *famous face flashing (FF), personally known FF,, & classic character flashing (CF*) | 9 | ALS (n=4),  MD (n=1),  SMA (n=2),  SBMA  (n=1) | M=50.0 SD=15.2  R=25-72 | M=13.8 SD=  10.2  R=2-28 years | Varied; described according to motor and speech function | N/R | N/R | With 1 sequence:  *Famous FF*  M=85.7  R=40-100  *Personal FF*  M=77.1  R=20-100  *CF*  M=11.4  R=0-40 | *1 seq.:*  5/9  *6+seq:*  9/9 | Bit rate with 1 sequence (bits/min):  *Famous FF*  M=52  *Personal FF*  M=45  *CF*  M=3 |
| Kleih et al., 2016 | Matrix speller | Copy-spell  (word) | N/A | 5 | CVA | M=58.6 SD=14.5R=46-83 | M=13.2 SD=  13.5  R=2-36 months | Aphasia severity: mild to medium (n=1), medium to severe (n=2), severe (n=2) | N/R | Ex: unable to give informed consent (n=1); Wi: after first session (n=1) | (n=4)  M=75.1 SD=32.7  R=30-100 | 3/4 | N/R |
| Krausz, Ortner, & Opisso, 2011 | Matrix speller | Copy-spell  (word) | N/A | 10 | CVA (n=2),  SCI (n=8) | M=35.6 SD=12.0  R=21-60 | N/R | ASIA:  A (n=6),  B (n=2);  LIS unspecified  (n=2) | N/R | N/R | M=62.0 SD=40.5  R=0-100 | 6/10 | NR |
| Mainsah et al., 2015 | Matrix speller | Copy-spell  (word) | 3 conditions:  *static stopping (S), dynamic stopping (D), & dynamic stopping with language model (LM)* | 10 | ALS | M=54.7 SD=8.3  R=38-63 | M=6.85 SD=4.5  R=1-16 years | ALSFRS-R: M=22.1 SD=14.5  R=1-42 | N/R | N/R | *Static*  M=79.4 SD=30.0  R=20-100  *Dynamic*  M=75.4 SD=27.2  R=20-97  *LM*  M=76.4 SD=25.6 R=22-100 | 7/10 | Bit rate (bits/min):  *Static*  M=6.4  SD=3.21  *Dynamic*  M=17.1 SD=11.78  *LM*  M=25.2 SD=19.56 |

| **Study** | **Interface**  **description** | **Task**  **(target)** | **Conditions or sessions** | **Sample**  **size** | **Diagnosis** | **Age**  **(years)** | **Time**  **since dx** | **Level of impairment** | **n with prior BCI exp.** | **Excluded/ withdrew, reason** | **Accuracy results (%)** | **n with**  **≥70%**  **accuracy** | **Other**  **measures** |
| --- | --- | --- | --- | --- | --- | --- | --- | --- | --- | --- | --- | --- | --- |
| ***EEG, ERP, Visual (continued)*** | | | | | | | | | | | | | |
| Mak et al., 2012 | Matrix speller | Copy-spell  (word, character) | N/A | 20 | ALS | M=56.9 SD=8.6 | N/R | ALSFRS:  M=6.9 SD=6.9;  Incomplete/ classic LIS (n=14) | N/R | N/R | M=57.1 SD=45.0  R=0-100 | 10/20 | N/R |
| Marchetti et al., 2013 | 4-choice icons | Icon selection | N/A | 10 | ALS | M=56.3 SD=7.8  R=44-68 | M=23.9 SD=26.4  R=6-96 months | ALSFRS-R: R=10-42 | N/R | N/R | *Day 1*  M=64.9  SD=6.1  *Day 2*  M=68.6 SD=4.9  *Day 3*  M=70.8  SD=5.1  *Day 4*  M=71.4  SD=6.4 | N/R | ITR (bits/min):  *Day 1*  M=3.3  SD=1.6  *Day 2*  M=3.8  SD=1.7  *Day 3*  M=4.9  SD=2.1  *Day 4*  M=6.3  SD=3.1 |
| McCane et al., 2014 | Matrix speller | Copy-spell  (word) | N/A | 25 | ALS | M=55.8 SD=8.6  R=41-72 | N/R | ALSFRS-R: M=6.2  SD=8.1  R=0-25 | 0/25 | N/R | PWD>70% accuracy  n=17 M=91.2 SEM=2.5 PWD<40% accuracy  M=12.3 SEM=6.2  R=0-36 | 17/25 | N/R |
| McCane et al., 2015 | Matrix speller | Copy-spell  (word) | N/A | 14 | ALS | M=55.9 SD=9.4  R=41-72 | N/R | ALSFRS-R:  M=9.4  SD=9.5  R=0-25 | 13/14 | N/R | M=95.7  SEM=2 | 14/14 | Characters per minute (cpm):  M=2.1  SEM=0.3  Bit rate (bits/min)  M=11.2  SE=1.3 |

| **Study** | **Interface**  **description** | **Task**  **(target)** | **Conditions or sessions** | **Sample**  **size** | **Diagnosis** | **Age**  **(years)** | **Time**  **since dx** | **Level of impairment** | **n with prior BCI exp.** | **Excluded/ withdrew, reason** | **Accuracy results (%)** | **n with**  **≥70%**  **accuracy** | **Other**  **measures** |
| --- | --- | --- | --- | --- | --- | --- | --- | --- | --- | --- | --- | --- | --- |
| ***EEG, ERP, Visual (continued)*** | | | | | | | | | | | | | |
| Medina-Juliá et al, 2020 | Matrix speller | Copy-spell (word & number) | 3 conditions:  *small,* m*edium*, & *large* *matrix* | 8 | ALS (n=7),  MD (n=1) | M=59.6 SD=17.0 | M=8.0 SD=6.9years | ALSFRS-R: R=0-31 | 0/8 | Ex: did not achieve system control (n=2) | *Small*  M=68.8  SD=30.5  *Medium:* M=75 SD=26.4 *Large:* M=67.7  SD=30.7 | *Small:*  4/8  *Medium:* 5/8 *Large:* 5/8 | N/R |
| Miao et al., 2020 | *Matrix speller & peripheral distribution speller* | Character selection | N/A | 18 | ALS | M=55.1 SD=12.9  R=30-70 | M=27.7 SD=15.1R=10-56 months | ALSFRS-R:  M=34.4  SD=8.6  R=19-47;  ALS stage:  2A (n=3), 2B (n=8),  3 (n=7) | 1/18 | N/R | *Matrix* M=82.0  SD=11.5  R=59.5-100  *Peripheral* M=83.5  SD=14.2, R=47.6-100 | *Matrix* 15/18  *Peripheral*  15/18 | ITR (bits/min):  *Matrix*  M=37.7  SD=11.4  R=19.2-60.2 *Peripheral*  M=39.0  SD=12.1  R=12.1-62.2 |
| Nam, Woo, & Bahn, 2012 | Matrix speller | Copy-spell  (phrase) | N/A | 9 | ALS (n=3),  CP (n=6) | M=41.2  SD=11.3 | N/R | N/R | N/R | N/R | N/R | N/R | ITR (bits/min)  M=0.7  SD=0.91 |
| Neshige et al., 2007 | *4-choice words & hiragana matrix speller* | Multiple-choice response selection (fx4), character selection, icon selection | N/A | 6 | ALS (n=6),  OPCA  (n=1) | M=60.5 SD=12.1 R=48-80 | N/R | Moderate-to-severe dysarthria/ mechanical ventilation; quadriplegia (n=3),  incomplete paralysis (n=1), use of all limbs (n=1) | N/R | N/R | *Word (n=5)*  M=100  *Character (n=3)*  M=100 | *Word*  5/6 *Character* 3/4 | N/R |

| **Study** | **Interface**  **description** | **Task**  **(target)** | **Conditions or sessions** | **Sample**  **size** | **Diagnosis** | **Age**  **(years)** | **Time**  **since dx** | **Level of impairment** | **n with prior BCI exp.** | **Excluded/ withdrew, reason** | **Accuracy results (%)** | **n with**  **≥70%**  **accuracy** | **Other**  **measures** |
| --- | --- | --- | --- | --- | --- | --- | --- | --- | --- | --- | --- | --- | --- |
| ***EEG, ERP, Visual (continued)*** | | | | | | | | | | | | | |
| Nijboer et al., 2008 | Matrix speller | Copy-spell  (sentence) | Phase I: 10 sessions over 6-14 weeks;  Phase II:10+  sessions over 17-40 weeks | 6 | ALS | M=48.5 SD=11.1R=36-67 | M=3.2 SD=2.6  R=1-8 years | ALSFRS-R:  M=12.3 SD=6.2  R=4-20 | N/R | Ex: did not achieve system control (n=1);  Wi: excessive setup time due to electrical noise (n=1) | *Phase I* M=62.0  *Phase II*  M=78.8 R=69.6-91.6 | *Phase I*  2/6  *Phase II*  3/4 | ITR (bits/min):  *Phase I*:  M=3.2  SD=3.6  *Phase II:*  M=9.7  SD=13.4;  Characters per minute (cpm):  *Phase I*  M=1.2  SD=1.4  *Phase II*  M=2.1  SD=3.3 |
| Oken et al., 2014 | Rapid serial visual presentation (RSVP) | Copy-spell  (word) | 5 levels (*L1-L5*) of word stimuli based on difficulty of target from least to most | 9 | ALS (n=4), MD (n=1), CVA (n=2), CP (n=1), AVM (n=1) | M=45.8  R=27-65 | M=14.8  R=1-55 | Incomplete LIS (n=1), classic LIS (n=2), total LIS (n=1) | N/R | Ex: did not pass cognitive screening (n=1); Wi: electrode problems or changes in medical status (n=2) | *L1 (n=6)*  6/6 complete M=90.9  R=33.3-100  *L2 (n=6)*  4/6 complete M=90.7 R=28.6-100  *L3 (n=4)*  2/4 complete  M=95.9  R=75.0-100  *L4 (n=2)*  1/2 complete M=100 R=100-100  *L5 (n=1)*  1/1 complete M=84.6 R=42.9-100 | N/R | Correct characters per minute (ccpm):  *L1* (n=6)  M=1.8  R=0.9-3.7  *L2* (n=6)  M=1.5  R=0.0-2.5  *L3* (n=4)  M=0.4  R=0.0-0.9  *L4* (n=2)  M=3.3  R=3.3-3.3  *L5* (n=1)  M=2.3  R=1.6-3.0 |

| **Study** | **Interface**  **description** | **Task**  **(target)** | **Conditions or sessions** | **Sample**  **size** | **Diagnosis** | **Age**  **(years)** | **Time**  **since dx** | **Level of impairment** | **n with prior BCI exp.** | **Excluded/ withdrew, reason** | **Accuracy results (%)** | **n with**  **≥70%**  **accuracy** | **Other**  **measures** |
| --- | --- | --- | --- | --- | --- | --- | --- | --- | --- | --- | --- | --- | --- |
| ***EEG, ERP, Visual (continued)*** | | | | | | | | | | | | | |
| Ortner et al., 2011 | Matrix speller | Copy-spell  (target unspeci-fied) | N/A | 15 | CVA (n=3),  SCI (n=8),  MS (n=3),  MD (n=1) | M=45.2 SD=17.1  R=21-68 | M=83.5 SD=98.5R=0-267  months | ASIA:  A (n=6)  B (n=2);  ALSFRS-R: 10 (n=1);  EDSS:  3 (n=2)  8 (n=1); Rankin Scale:  V (n=2)  IV (n=1). | N/R | N/R | M=70.7 SD=27.1  R=0-100 | 10/15 | N/R |
| Pires, Nunes, Castelo-Branco, 2011 | Matrix speller | Copy-spell  (sentence) | N/A | 5 | ALS (n=3),  CP (n=2) | M=48.0 SD=23.4  R=18-75 | ALS  R=1-7 years  CP  lifetime | ALS  ALSFRS-R:  M=43;  CP  spastic tetraparesis & dysarthria | 0/5 | Ex: did not achieve system control (n=1, excluded from ALS group results) | CP  M=96.7  ALS (n=2)  M=96.9 | 4/5 | Symbols/min (spm):  CP  M=3.1  ALS  M=3.8  Bandwidth (bits/min):  CP  M=15.1  ALS  M=18.2 |

| **Study** | **Interface**  **description** | **Task**  **(target)** | **Conditions or sessions** | **Sample**  **size** | **Diagnosis** | **Age**  **(years)** | **Time**  **since dx** | **Level of impairment** | **n with prior BCI exp.** | **Excluded/ withdrew, reason** | **Accuracy results (%)** | **n with**  **≥70%**  **accuracy** | **Other**  **measures** |
| --- | --- | --- | --- | --- | --- | --- | --- | --- | --- | --- | --- | --- | --- |
| ***EEG, ERP, Visual (continued)*** | | | | | | | | | | | | | |
| Pires, Nunes, Castelo-Branco, 2012 | *Matrix speller & lateral single character speller (LSC)* | Copy-spell  (sentence) | N/A | 14 | ALS (n=7),  MD (n=1),  CP (n=5),  SCI (n=1) | M=53.2 SD=21.2  R=18-80 | ALS  R=0-7 years  SCI  12 years MD  22 years; CP lifetime | ALS  ALSFRS-R: M=41.4 SD=5.0  R=32-47;  Other Dx  tetraparesis/ tetraplegia | N/R | Ex: did not achieve system control (*Matrix* n=5, *LSC* n=6, excluded from condition results) | *Matrix*  ALS (n=6)  M=91.7  Other Dx (n=4)  M=85.4  *LSC*  ALS (n=5)  M=91.3  Other Dx (n=3)  M=81.6 | *Matrix*  10/14  *LSC*  8/14 | Symbols per minute (spm)  *Matrix*  ALS  M=4.1  Other Dx  M=3.3  *LSC*  ALS  M=5.0  Other Dx  M=3.5  ITR (bits/min):  *Matrix*  ALS  M=17.8  Other Dx  M=13.0  *LSC*  ALS  M=20.0  Other Dx  M=11.6 |
| Riccio et al., 2013 | Matrix speller | Copy-spell  (word) | N/A | 8 | ALS | M=58.3 SD=12.3 R=40-75 | N/R | ALSFRS-R: M=31.8 SD=8.6  R=13-41 | 0/8 | Ex: other medical conditions or lack of communi-cation channel (n=N/R) | M=97.5  SD=3.8  R=90-100 | 8/8 | N/R |
| Riccio et al., 2015 | Matrix speller | Copy-spell  (word) | 2 conditions: *with or without EMG for error correction* | 3 | ALS (n=1),  CVA (n=2) | M=50.3 SD=3.2 R=48-54 | N/R | Tetraparesis/ hemiplegia; dysarthria/ anarthria | 1/3 | N/R | *With EC*  M=97.3  R=92-100, *Without EC*  M=73.7  R=63-93 | *With EC*  3/3  *Without*  1/3 | ITR (bits/min):  *With EC*  Med=7.2  R=5.9-12.5 *Without EC*  Med=4.1  R=1.5-5.0 |

| **Study** | **Interface**  **description** | **Task**  **(target)** | **Conditions or sessions** | **Sample**  **size** | **Diagnosis** | **Age**  **(years)** | **Time**  **since dx** | **Level of impairment** | **n with prior BCI exp.** | **Excluded/ withdrew, reason** | **Accuracy results (%)** | **n with**  **≥70%**  **accuracy** | **Other**  **measures** |
| --- | --- | --- | --- | --- | --- | --- | --- | --- | --- | --- | --- | --- | --- |
| ***EEG, ERP, Visual (continued)*** | | | | | | | | | | | | | |
| Ron-Angevin, Varona-Moya, & da Silva-Sauer, 2015 | *Matrix speller & T9 speller* | Copy-spell  (sentence) | N/A | 1 | ALS | 62 | N/R | Incomplete  LIS | 1/1 | N/R | *Matrix*  93.3  *T9*  88.2 | 1/1 | Time to copy-spell 7-word sentence (s):  *Matrix*  1035  *T9*  663 |
| Schettini et al., 2015 | Matrix speller | Copy-spell  (word) | 2 conditions:  *P300 speller (P300) & AT prototype (ATP)* | 8 | ALS | M=60.1 SD=12.6 R=40-75 | M=24.1  SD=26.7  R=2-84  months | ALSFRS-R: M=32.0 SD=8.7  R=13-41 | N/R | N/R | *P300*  M=97.5  SD=3.8  R=90-100  *ATP*  M=90.9  SD=10.7  R=84.5-100 | *P300*  8/8  *ATP*  7/8 | Seconds/ correct selection (s):  *ATP*  M=31.7  SD=7.6  Correct selections/ minute (cspm):  *ATP*  M=1.9 |
| Sellers, Vaughan, & Wolpaw,  2010 | Matrix speller | Copy-spell  (target unspeci-fied) | 1-2 session per week over 2.5 yrs | 1 | ALS | 51 | N/R | ALSFRS-R: 1 | No | N/R | Med=83 | 1/1 | N/R |
| Sellers, Ryan, & Hauser, 2014 | *Matrix speller, 4-choice words, & multi-step speller* | Yes/no questions,  copy-spell  (word) | 62 sessions on 34 days over 56 weeks, multiple system configura-tions | 1 | CVA | 68 | 7 months at study start | LIS unspecified type | N/R | N/R | *6x6 matrix*  M=72  *4x4 matrix*  N/R  *4-choice*  M=94.7  *7-choice*  M=84.6  *3x5 matrix*  M=77.8  *3x6 matrix*  M=81.5 | 1/1 | N/R |

| **Study** | **Interface**  **description** | **Task**  **(target)** | **Conditions or sessions** | **Sample**  **size** | **Diagnosis** | **Age**  **(years)** | **Time**  **since dx** | **Level of impairment** | **n with prior BCI exp.** | **Excluded/ withdrew, reason** | **Accuracy results (%)** | **n with**  **≥70%**  **accuracy** | **Other**  **measures** |
| --- | --- | --- | --- | --- | --- | --- | --- | --- | --- | --- | --- | --- | --- |
| ***EEG, ERP, Visual (continued)*** | | | | | | | | | | | | | |
| Silvoni et al., 2013 | 4-choice icons | icon selection | 4 sessions over 3 years | *Session 1:*  n=24  *Session 2:* n=9  *Session 3:* n=5  *Session 4:* n=3 | ALS | M=56 SD=14  at start  of study | M=46  SD=31  months | ALSFRS-R: M=32  SD=7  at study start | N/R | Wi: death (n=2),  other (n=19) | *Session 1*  M=87.5  SD=18.1  *Session 2*  M=72.9  SD=21.9  *Session 3*  M=72.5  SD=22.4  *Session 4*  M=79.2  SD=26 | *Session 1*  21/24  *Session 2*  6/9  *Session 3*  4/5  *Session 4*  2/3 | ITR (bits/min):  *Session 1*  M=6.6  SD=1.78  *Session 2*  M=6.6  SD=2.63  *Session 3*  M=7.1  SD=2.77  *Session 4*  M=6.5  SD=1.42 |
| Spuler et al., 2012 | Matrix speller | Copy-spell  (target unspeci-fied) | 2 conditions;  *with* & *without error*  *correction* | 6 | ALS (n=3),  MD (n=1) | M=51.2 SD=10.2 R=36-63 | N/R | ALSFRS-R:  M=23.5 SD=14.9  R=8-43 | N/R | Ex: fatigue & difficulty  following  directions (n=1) | N/R | N/R | Bit rate  (bits/min):  *With error correction*  M=2.1  SD=1.07 |
| Townsend  et al., 2010 | Matrix speller | Copy-spell  (word & #  string) | 2 conditions:  *Checker-board paradigm (CBP) &*  *row-column*  *paradigm (RCP)* | 3 | ALS | N/R | N/R | Incomplete  LIS (n=1), classic LIS (n=2) | 3/3 | N/R | *CBP*  M=84  R=78-89  *RCP*  M=59  R=46-68 | *CBP*  n=3/3  *RCP*  n=0/3 | Practical bit rate (bits/min):  *CBP*  M=8.9  SD=5.19  R=3.3-13.5 |

| **Study** | **Interface**  **description** | **Task**  **(target)** | **Conditions or sessions** | **Sample**  **size** | **Diagnosis** | **Age**  **(years)** | **Time**  **since dx** | **Level of impairment** | **n with prior BCI exp.** | **Excluded/ withdrew, reason** | **Accuracy results (%)** | **n with**  **≥70%**  **accuracy** | **Other**  **measures** |
| --- | --- | --- | --- | --- | --- | --- | --- | --- | --- | --- | --- | --- | --- |
| ***EEG, ERP, Visual (continued)*** | | | | | | | | | | | | | |
| Wolpaw et al., 2018 | Matrix speller | Copy-spell  (target unspeci-fied) | Not all participants used BCI in their homes | 39 | ALS | All  n=39  M=61  SD=12  Home users  n=14  M=58  SD=12 | All  n=39  M=6.1 SD=5.0 years  Home users  n=14  M=6.9 SD=5.4 years | ALSFRS-R:  All  n=39  M=13.8 SD=12.3  Home users  n=14  M=16.4  SD=12.8 | N/R | Ex: did not meet inclusion criteria (n=3), did not meet initial BCI criterion (n=9); Wi: death or illness (n=14), limitations of system (n=4), lost to follow up (n=1), other (n=2) | Home users  M=73  SD=11 | 28/39 | Selections per minute (spm)  Home users  M=2.9  R=1.3 |
| Zickler et al., 2011 | Matrix speller | Copy-spell  (word) | N/A | 4 | ALS (n=1),  MD (n=2),  SMA (n=1) | M=45.3 SD=8.7  R=37-55 | N/R | Described as “severely disabled” & “reliant on AT” | 3/4 | N/R | M=96.4  SD=6.2  R=85.7-100 | 4/4 | ITR (bits/min):  M=7.5  SD=0.82  R=6.05-7.95 |
| Zickler et al., 2013 | Matrix speller | Copy-spell  (non-word character) | N/A | 4 | ALS (n=2),  MD (n=1),  CVA (n=1) | M=48.3 SD=7.6  R=39-55 | N/R | Described as “severely disabled” & “reliant on AT” | N/R | N/R | M=90  SD=7.1  R=80-95 | 4/4 | ITR (bits/min):  M=5.6  SD=0.74  R=4.56-6.13 |
| ***EEG, ERP, Auditory*** | | | | | | | | | | | | | |
| Halder, Käthner, & Kübler, 2016a | Auditory with visual aid and spatial cues, animal sounds | Copy-spell  (word) | 5 sessions | 5 | ALS (n=2), MD (n=1), anoxic TBI (n=1), MS (n=1) | M=61.2 SD=14.3R=45-79 | M=14.4 SD=  11.4  years | ALSFRS-R: M=27.0 SD=8.0 | 0/5 | N/R | *Session 1* M=11.2  *Session 5*  M=52.8 | By session 5: 2/5 | ITR (bits/min):  *Session 1*  M=0.2  *Session 5*  M=3.1 |

| **Study** | **Interface**  **description** | **Task**  **(target)** | **Conditions or sessions** | **Sample**  **size** | **Diagnosis** | **Age**  **(years)** | **Time**  **since dx** | **Level of impairment** | **n with prior BCI exp.** | **Excluded/ withdrew, reason** | **Accuracy results (%)** | **n with**  **≥70%**  **accuracy** | **Other**  **measures** |
| --- | --- | --- | --- | --- | --- | --- | --- | --- | --- | --- | --- | --- | --- |
| ***EEG, ERP, Auditory (continued)*** | | | | | | | | | | | | | |
| Halder et al., 2016b | Auditory with visual aid and spatial cues, syllables for selection of consonant and vowel in hiragana | Character selection | 3 sessions | 1 | SCI | 43 | 23 years | N/R | N/R | N/R | *Session 1*  12  *Session 2*  28  *Session 3*  56 | 0/1 | ITR (bits/min):  *Session 1*  0.2  *Session 2*  0.7  *Session 3*  2.0 |
| Hill et al., 2014 | Auditory, words | Yes/no questions | N/A | 2 | ALS | M=58.5 | 14, 15 years | Incomplete LIS (n=1), classic LIS (n=1) | 2/2 | N/R | M=84.4 | 2/2 | N/R |
| Kleih et al., 2015 | Auditory, words containing target letters | Copy-spell  (word | N/A | 4 | ALS (n=1),  MD (n=1), traumatic accident (n=2) | M=55.5 SD=12.6R=43-72 | M=17.0 SD=17.4R=2-42 months  at start of study | Classified as: minor  (n=1), moderate (n=1), major  (n=2) | N/R | N/R | M=74.2 SD=15.0  R=51.9-84.2 | 3/4 | ITR (bits/min):  M=1.3  SD=0.8 |
| Kübler et al., 2009 | Auditory with visual aid, numbers | Copy-spell  (word) | N/A | 4 | ALS | M=44.5 SD=6.4  R=39-51 | M=6.3 SD=3.3  R=3-10 years | Incomplete LIS (n=2), incomplete LIS with periods of total LIS (n=1), tetraplegia and severe dysarthria (n=1) | 4/4 | N/R | M=12.1  SD=14.02 R=0-25 | 0/4 | N/R |
| Lulé et al., 2013 | Auditory, words | Yes/no questions | N/A | 18 | CVA (n=2), DOC  s/p anoxic injury  (n=2), DOC s/p TBI (n=5), DOC unspecified etiology (n=9) | LIS  R=29-63 MCS  M=42 SD=21  UWS  M=61  SD=17 | LIS  R=26-46 months  MCS  M=70 SD=109months UWS  M=10  SD=15 months | LIS unspecified  (n=2), MCS  (n=13), UWS (n=3) | N/R | N/R | LIS  M=40.0  SD=28.3  R=20-60  MCS  M=26.8 SD=14.1  R=0-50  UWS  M=33.3 SD=5.8  R=30-40 | 0/18 | N/R |

| **Study** | **Interface**  **description** | **Task**  **(target)** | **Conditions or sessions** | **Sample**  **size** | **Diagnosis** | **Age**  **(years)** | **Time**  **since dx** | **Level of impairment** | **n with prior BCI exp.** | **Excluded/ withdrew, reason** | **Accuracy results (%)** | **n with**  **≥70%**  **accuracy** | **Other**  **measures** |
| --- | --- | --- | --- | --- | --- | --- | --- | --- | --- | --- | --- | --- | --- |
| ***EEG, ERP, Auditory (continued)*** | | | | | | | | | | | | | |
| Onishi et al., 2017 | Auditory, positive and negative affective sounds | Yes/no questions | N/A | 1 | ALS | 61 | N/R | ALSFRS-R:  0 | N/R | N/R | 90 | 1/1 | N/R |
| Simon et al., 2015 | Auditory with visual aid, animal sounds | Copy-spell  (word) | 2 sessions over 2 days | 1 | ALS | 66 | 3 years | ALSFRS-R: 17.5 at study start, 16 one month later | 0/1 | N/R | *Session 1* M=20  SD=9.7  *Session 2* M=47 SD=20.1 | 0/1 | Bit rate (bits/min):  *Session 1*  M=0.3  *Session 2*  M=1.4 |
| Schreuder et al., 2013 | Auditory with visual aid and spatial cues, 2-step letter selection | Copy-spell  (target unspeci-fied) | N/A | 1 | CVA | 48 | 4 years | Incomplete LIS | 1/1 | N/R | Best within-session accuracy  M=39 | 0/1 | N/R |
| ***EEG, ERP, Audiovisual*** | | | | | | | | | | | | | |
| Wang et al., 2017 | Audiovisual | Yes/no questions | N/A | 13 | DOC s/p TBI (n=4),  DOC s/p injury  (n=9) | M=37.2 SD=12.4  R=17-59 | M=2.6 SD=2.0  R=1-8.5 months | JFK CRS-R: M=7.2 SD=2.5  R=4-14;  VS (n=8), MCS (n=5) | N/R | N/R | M=69.1 SD=12.3  R=44.4-86.5 | 7/13 | N/R |
| ***EEG, ERP, Tactile*** | | | | | | | | | | | | | |
| Lugo et al., 2014 | Tactile, 3 tactors | Yes/no questions | N/A | 6 | CVA | M=40.2 SD=10.9R=21-48 | M=10.0 SD=6.6 years | LIS unspecified | 5/6 | N/R | M=55.3 SD=27.3  R=20-100 | 1/6 | N/R |
| ***EEG, ERP, Visual, auditory, & audiovisual*** | | | | | | | | | | | | | |
| Sellers et al., 2006 | *Visual, 4-choice words, auditory, 4-choice words, & audiovisual (AV), 4-choice words* | Yes/no questions, binary-choice response selection | N/A | 3 | ALS | M=43.7 SD=6.5  R=37-50 | N/R | Described as "able to communi-cate" and either able to use upper extremities (n=2) or with only head move-ment and some speech (n=1) | N/R | N/R | *Visual*  M=63.4  SD=10.0  R=53.9-73.8 *Auditory*  M=65.9  SD=7.1  R=59.1-73.2  *AV*  M=65.1  SD=3.7  R=61.6-64.9 | *Visual*  1/3  *Auditory* 1/3  *AV*  0/3 | N/R |

| **Study** | **Interface**  **description** | **Task**  **(target)** | **Conditions or sessions** | **Sample**  **size** | **Diagnosis** | **Age**  **(years)** | **Time**  **since dx** | **Level of impairment** | **n with prior BCI exp.** | **Excluded/ withdrew, reason** | **Accuracy results (%)** | **n with**  **≥70%**  **accuracy** | **Other**  **measures** |
| --- | --- | --- | --- | --- | --- | --- | --- | --- | --- | --- | --- | --- | --- |
| ***EEG, ERP, Visual & tactile*** | | | | | | | | | | | | | |
| Severens et al., 2014 | *Visual, multi-step speller & tactile with visual aid, 5-tactor multi-step speller* | Copy-spell  (target unspeci-fied) | N/A | 5 | ALS | M=39 SD=13.8R=23-56 | M=1.3 SD=0.8 R=0.3-2.4 years | ALSFRS-R (n=4):  M=34.8 SD=10.8  R=24-45 | N/R | N/R | *Tactile*  M=53  *Visual*  M=85 | *Tactile*  1/5  *Visual*  4/5 | ITR (bits/min):  *Tactile*  M=6.6  *Visual*  M=8.7 |
| Kaufmann, Holz, & Kübler, 2013a | *Visual, matrix speller & RSVP; tactile with visual aid, 4-tactor multi-step speller* | Character selection & copy-spell (word) | N/A | 1 | CVA | 46 | 7 years | Incomplete LIS | 0/1 | No | *Tactile*  M=50  *Visual*  Visual paradigms did not elicit classifiable ERPs | 0/1 | N/R |
| ***EEG, ERP & SSVEP, Visual*** | | | | | | | | | | | | | |
| Combaz et al., 2013 | *Visual, matrix speller (ERP) & multi-step speller (SSVEP)* | Free-spell (word) | N/A | 7 | CVA (n=6),  TBI (n=1) | M=43.0 SD=12.1R=21-61 | M=7.5 SD=5.8  R=1.2-17.5 years | ALSFRS-R:  M=20.7 SD=5.3  R=14-30 | N/R | Ex: visual impairment or attention deficit (n=3); Wi: severe depression (n=1) | *ERP*  M=61.3  SD=18.9 R=37.5-85.85  *SSVEP* M=77.3 SD=12.7  R=56.2-94.6 | *ERP*  3/7  *SSVEP*  7/7 | ITR (bits/min):  *ERP*  M=6.0  SD=4.92  R=1.6-15.2 *SSVEP*  M=13.1 SD=5.58  R=7.4-22.9 |
| ***EEG, ERP & MI, Tactile (ERP) & task instructions only (MI)*** | | | | | | | | | | | | | |
| Guger et al., 2017 | *Tactile, 3 tactors (ERP) & task instructions only (MI)* | Yes/no questions | N/A | 12 | ALS | M=62.2 SD=11.2  R=37-76 | M=94.4 SD=  50.3  R=21-184 months | Classic LIS (n=9), total LIS (n=3) | N/R | Ex (from use of one of the two systems): time constraints or fatigue (n=3 for tactile, n=9 for MI) | *Tactile (n=9)*  M=80.0 SD=8.7  R=70-90;  *MI (n=3)*  M=93.3 | *Tactile*  9/12  *MI*  3/10 | N/R |

| **Study** | **Interface**  **description** | **Task**  **(target)** | **Conditions or sessions** | **Sample**  **size** | **Diagnosis** | **Age**  **(years)** | **Time**  **since dx** | **Level of impairment** | **n with prior BCI exp.** | **Excluded/ withdrew, reason** | **Accuracy results (%)** | **n with**  **≥70%**  **accuracy** | **Other**  **measures** |
| --- | --- | --- | --- | --- | --- | --- | --- | --- | --- | --- | --- | --- | --- |
| ***EEG, MI*** | | | | | | | | | | | | | |
| Leeb et al., 2013 | Visual, multi-step speller | Copy-spell  (word) | N/A | 24 | ALS (n=2), MD (n=1), CP (n=1), SCI (1), myopat-hy (n=4), tetraplegia s/p Guillain Barre (n=1)  tetra-plegia (n=10), iLIS (n=1), amputation (n=1), SCA (n=1), left shoulder-hand syndrome (n=1) | M=42.7 SD=14.1 R=18.4-70.2 | R=1.2-36.8 years | N/R | 0/24 | Ex: technical difficulties (n=1), muscle artifacts (n=2), did not achieve system control (n=10); Wi (n=5) | (n=6)  M=93  SD=5 | 10/20 | Characters per minute (cpm):  Max=2 |
| Mangia et al., 2014 | Task instructions only, two mental states used for binary-choice responses | Yes/no questions | N/A | 5 | DOC s/p TBI | M=42.0 SD=18.5R=22-63 | M=11.6 SD=7.8  R=4-22 months | Conscious state (n=1),  MCS (n=3), VS/MCS (n=1);  Level of cognitive functioning: M=4  SD=1.7  R=3-7 | N/R | N/R | M=91.7 SD=7.4 | N/R | N/R |
| Neuper et al., 2003 | Visual, multi-step speller | Copy-spell  (word) | N/A | 1 | CP | 32 | 32 years | Severe spastic tetraparesis | N/R | N/R | N/R | N/R | Characters per minute (cpm), range for 2/week over 22 weeks:  R=0.2-2.5 |

| **Study** | **Interface**  **description** | **Task**  **(target)** | **Conditions or sessions** | **Sample**  **size** | **Diagnosis** | **Age**  **(years)** | **Time**  **since dx** | **Level of impairment** | **n with prior BCI exp.** | **Excluded/ withdrew, reason** | **Accuracy results (%)** | **n with**  **≥70%**  **accuracy** | **Other**  **measures** |
| --- | --- | --- | --- | --- | --- | --- | --- | --- | --- | --- | --- | --- | --- |
| ***EEG, MI (continued)*** | | | | | | | | | | | | | |
| Perdikis et al., 2014 | Visual, multi-step speller | Copy-spell  (word) | 3 conditions: *with EMG for error correction (hBCI), with context-aware data compresison (CA), and with both features (hBCI+CA)* | 6 | MD (n=1),  SCI (n=5) | M=39.0 SD=15.5  R=19-60 | N/A | Tetraplegia (n=5), unspecified (n=1) | 6/6 | Ex: did not meet initial BCI criterion (n=N/R); Wi (n=N/R) | Word completion success rate:  *hBCI+CA (n=6)*  100  *hBCI (n=6)*  95.8  *CA (n=2)*  100 | 6/6 | For all participants (not reported separately for PWD):  Seconds per character (spc):  *hBCI+CA*  M=35.7  *hBCI*  M=44.9  *CA*  M=34.2  Characters per minute (cpm):  *hBCI+CA*  M=1.7  *hBCI*  M=1.3  *CA*  M=1.8 |
| Scherer et al., 2015 | Visual, icon matrix with row-column scanning | Icon selection | N/A | 12 | CP | M=35.4 SD=11.5  R=20-56 | Lifetime | GMFCS: IV (n=4), V (n=7) | N/R | Ex: technical difficulties, EEG signal quality (n=3) | M=19.5  SD=22.5  R=0-60 | 0/11 | N/R |
| ***EEG, SCP, Task instructions only*** | | | | | | | | | | | | | |
| Hinterber-ger, Bir-baumer, & Flor, 2005 | Task instructions only with auditory feedback, positive and negative potential shifts used for binary-choice responses | Yes/no questions & multiple-choice questions with yes/no for each response option | N/A | 1 | ALS | 58 | 8 years | Total LIS | N/R | N/R | M=65 | 0/1 | N/R |

| **Study** | **Interface**  **description** | **Task**  **(target)** | **Conditions or sessions** | **Sample**  **size** | **Diagnosis** | **Age**  **(years)** | **Time**  **since dx** | **Level of impairment** | **n with prior BCI exp.** | **Excluded/ withdrew, reason** | **Accuracy results (%)** | **n with**  **≥70%**  **accuracy** | **Other**  **measures** |
| --- | --- | --- | --- | --- | --- | --- | --- | --- | --- | --- | --- | --- | --- |
| ***EEG, SCP, Visual*** | | | | | | | | | | | | | |
| Hinterber-ger et al., 2003 | Visual, multi-step speller | copy-spell (target unspeci-fied) | N/A | 1 | ALS | 46 | N/R | Incomplete LIS | 1/1 | No | M=72 | N/R | N/R |
| Kübler et al., 1999 | Visual, multi-step speller | Copy-spell  (word) | N/A | 3 | ALS | M=43.0 SD=6.6  R=37-50 | M=9.7 SD=4.5  R=5-14 years | Incomplete LIS (n=1), classic LIS (n=1), severe tetraparesis and dysarthria (n=1) | N/R | N/R | N/R | N/R  2/3 PWD achieved system control | (n=2)  Seconds per letter (spl):  M=64.7  SD=41.3  R=12-192  Characters per minute (cpm):  M=0.9  R=0.3-5.0 |
| Neumann et al., 2003 | Visual, multi-step speller | Free-spell  paragraph | N/A | 1 | ALS | N/R, ~41 at start of 4 year study | 7 years at start of study | Incomplete LIS | 0/1 | No | N/R | N/R | Typed 454 German words (4733 characters) in ~160 hours spread across 52 days over 6 months. Characters per minute (cpm; mean across sessions): ~2 |
| ***EEG, SSVEP, Visual*** | | | | | | | | | | | | | |
| Lesenfants et al., 2014 | Visual, binary choice color stimuli | Yes/no questions | N/A | 6 | CVA (n=5),  TBI (n=1) | M=49.0 SD=19.7  R=23-74 | R=3-15 years | LIS unspecified | 0/6 | Wi: fatigue (n=2) | (n=4)  M=59.5  SD=9.0 | 1/4 | N/R |
| Peters et al., 2020 | Visual, multi-step speller | Copy-spell (word) | N/A | 2 | ALS | M=56 | 7; 12 years | ALSFRS-R:  0 | 2/2 | N/R | M=61  R=0-100 | 0/2 | Correct characters per minute (ccpm):  M=0.3 |

| **Study** | **Interface**  **description** | **Task**  **(target)** | **Conditions or sessions** | **Sample**  **size** | **Diagnosis** | **Age**  **(years)** | **Time**  **since dx** | **Level of impairment** | **n with prior BCI exp.** | **Excluded/ withdrew, reason** | **Accuracy results (%)** | **n with**  **≥70%**  **accuracy** | **Other**  **measures** |
| --- | --- | --- | --- | --- | --- | --- | --- | --- | --- | --- | --- | --- | --- |
| ***fNIRS, Task instructions only*** | | | | | | | | | | | | | |
| Abdalma-lak et al., 2017 | Task instructions only, two mental states used for binary-choice responses | Yes/no questions | N/A | 1 | Guillain-Barre | 75 | N/R | Classic LIS | N/R | N/R | M=100 | 1/1 | N/R |
| Gallegos-Ayala et al., 2014 | Task instructions only, two mental states used for binary-choice responses | Yes/no questions | N/A | 1 | ALS | 67 | 5 years | ALSFRS-R: 0; total LIS | 1/1 | N/R | M=74.6 | 1/1 | NR |
| Naito et al., 2007 | Task instructions only, two mental states used for binary-choice responses | Binary-choice response selection | N/A | 40 | ALS | R=22-80 | N/R | Total LIS (n=17), unspecified but relying on mechanical ventilation (n=23) | N/R | Ex: did not achieve system control (n=17) | M=79.6 SD=13.0  R=60-100 | 32/40 | N/R |
| ***fNIRS, Visual*** | | | | | | | | | | | | | |
| Schudlo & Chau, 2018 | Visual, 3-choice icons | Multiple-choice questions | N/A | 1 | Congenital idiopathic weakness | 26 | 26 years | Control of eyes and facial muscles; limited, inconsistent control of arms and legs | N/R | N/R | M=63.3 | 0/1 | N/R |

| **Study** | **Interface**  **description** | **Task**  **(target)** | **Conditions or sessions** | **Sample**  **size** | **Diagnosis** | **Age**  **(years)** | **Time**  **since dx** | **Level of impairment** | **n with prior BCI exp.** | **Excluded/ withdrew, reason** | **Accuracy results (%)** | **n with**  **≥70%**  **accuracy** | **Other**  **measures** |
| --- | --- | --- | --- | --- | --- | --- | --- | --- | --- | --- | --- | --- | --- |
| ***Implantable*** | | | | | | | | | | | | | |
| Bacher et al., 2015 | Intracortical microelec-trode array, visual, onscreen keyboard with cursor control | Copy-spell (word & phrase) | N/A | 1 | CVA | 58 | 14 years | Tetraplegia; anarthria | 1/1 | N/R | *QWERTY* M=82.9  SD=9.7  *Radial*  M=91.4 SD=5.8 | 1/1 | Keystrokes per minute (kpm):  *QWERTY*  M=6.3  SD=1.0  *Radial*  M=12.7  SD=2.2 |
| Pandarinath et al., 2017 | Intracortical microelec-trode array, visual, onscreen keyboard with cursor control | Copy-spell  (sentence) | N/A | 3 | ALS (n=2),  SCI (n=1) | M=56.3 SD=6.1 R=51-63 | N/R | ALSFRS-R: 16 (n=1) or 17 (n=1); ASIA C (n=1) | N/R | N/R | N/R | N/R | Correct characters per minute (ccpm):  M=28.1;  ITR (bits/min):  M=144 |
| Vansteen-sel et al, 2016 | Subdural electrodes, visual, matrix speller | Copy-spell (word) | N/A | 1 | ALS | 58 | 7 years | ALSFRS-R: 2; classic LIS | N/R | Excluded, died before electrode implantation (n=2) | Across 44 spelling runs:  M=89  SD=6 | 1/1 | Seconds per letter (spl):  M=33  Characters per minute (cpm):  M=1.8 |

**References**

Abdalmalak, A., Milej, D., Norton, L., Debicki, D. B., Gofton, T., Diop, M., et al. (2017). Single-session communication with a locked-in patient by functional near-infrared spectroscopy. *Neurophotonics* 4(4), 040501. doi: 10.1117/1.NPh.4.4.040501.

Bacher, D., Jarosiewicz, B., Masse, N. Y., Stavisky, S. D., Simeral, J. D., Newell, K., et al. (2015). Neural point-and-click communication by a person with incomplete locked-in syndrome. *Neurorehabil. Neural Repair* 29(5), 462–471. doi: 10.1177/1545968314554624.

Carabalona, R., Grossi, F., Tessadri, A., Castiglioni, P., Caracciolo, A., and de Munari, I. (2012). Light on! Real world evaluation of a P300-based brain-computer interface (BCI) for environment control in a smart home. *Ergonomics* 55(5), 552–563. doi: 10.1080/00140139.2012.661083.

Caruso, M., Cincotti, F., Leotta, F., Mecella, M., Riccio, A., Schettini, F., et al. (2013). “My-World-in-My-Tablet: An Architecture for People with Physical Impairment,” in *Human-Computer Interaction. Interaction Modalities and Techniques*, ed. M. Kurosu (Berlin, Heidelberg: Springer Berlin Heidelberg), 637–647. doi: 10.1007/978-3-642-39330-3_69.

Clements, J. M., Sellers, E. W., Ryan, D. B., Caves, K., Collins, L. M., and Throckmorton, C. S. (2016). Applying dynamic data collection to improve dry electrode system performance for a P300-based brain-computer interface. *J. Neural Eng.* 13:066018. doi: 10.1088/1741-2560/13/6/066018.

Combaz, A., Chatelle, C., Robben, A., Vanhoof, G., Goeleven, A., Thijs, V., et al. (2013). A Comparison of Two Spelling Brain-Computer Interfaces Based on Visual P3 and SSVEP in Locked-In Syndrome. *PLoS ONE* 8, e73691. doi: 10.1371/journal.pone.0073691.

Cruz, A., Pires, G., and Nunes, U. J. (2018). Double ErrP Detection for Automatic Error Correction in an ERP-Based BCI Speller. *IEEE Trans. Neural Syst. Rehabil. Eng.* 26, 26–36. doi: 10.1109/TNSRE.2017.2755018.

Gallegos-Ayala, G., Furdea, A., Takano, K., Ruf, C. A., Flor, H., and Birbaumer, N. (2014). Brain communication in a completely locked-in patient using bedside near-infrared spectroscopy. *Neurology* 82, 1930–1932. doi: 10.1212/WNL.0000000000000449.

Geronimo, A., Simmons, Z., and Schiff, S. J. (2016). Performance predictors of brain-computer interfaces in patients with amyotrophic lateral sclerosis. *J. Neural Eng.* 13:026002. doi: 10.1088/1741-2560/13/2/026002.

Guger, C., Spataro, R., Allison, B. Z., Heilinger, A., Ortner, R., Cho, W., et al. (2017). Complete locked-in and locked-in patients: Command following assessment and communication with vibro-tactile P300 and motor imagery brain-computer interface tools. *Front. Neurosci.* 11, 256. doi: 10.3389/fnins.2017.00251.

Guy, V., Soriani, M. H., Bruno, M., Papadopoulo, T., Desnuelle, C., and Clerc, M. (2018). Brain computer interface with the P300 speller: Usability for disabled people with amyotrophic lateral sclerosis. *Ann. Phys. Rehabil. Med.* 61, 5–11. doi: 10.1016/j.rehab.2017.09.004.

Halder, S., Käthner, I., and Kübler, A. (2016a). Training leads to increased auditory brain-computer interface performance of end-users with motor impairments. *Clin. Neurophysiol.* 127, 1288–1296. doi: 10.1016/j.clinph.2015.08.007.

Halder, S., Takano, K., Ora, H., Onishi, A., Utsumi, K., and Kansaku, K. (2016b). An Evaluation of Training with an Auditory P300 Brain-Computer Interface for the Japanese Hiragana Syllabary. Front. Neurosci. 10, 446. doi: 10.3389/fnins.2016.00446.

Hill, N. J., Ricci, E., Haider, S., McCane, L. M., Heckman, S., Wolpaw, J. R., et al. (2014). A practical, intuitive brain-computer interface for communicating “yes” or “no” by listening. *J. Neural Eng.* 11:035003. doi: 10.1088/1741-2560/11/3/035003.

Hinterberger, T., Birbaumer, N., and Flor, H. (2005). Assessment of cognitive function and communication ability in a completely locked-in patient. *Neurology* 64, 1307–1308. doi: 10.1212/01.WNL.0000156910.32995.F4.

Hinterberger, T., Kübler, A., Kaiser, J., Neumann, N., and Birbaumer, N. (2003). A brain–computer interface (BCI) for the locked-in: comparison of different EEG classifications for the thought translation device. *Clin. Neurophysiol.* 114, 416–425. doi: 10.1016/S1388-2457(02)00411-X.

Ikegami, S., Takano, K., Kondo, K., Saeki, N., and Kansaku, K. (2014). A region-based two-step P300-based brain-computer interface for patients with amyotrophic lateral sclerosis. *Clin. Neurophysiol.* 125, 2305–2312. doi: 10.1016/j.clinph.2014.03.013.

Ikegami, S., Takano, K., Saeki, N., and Kansaku, K. (2011). Operation of a P300-based brain-computer interface by individuals with cervical spinal cord injury. *Clin. Neurophysiol.* 122, 991–996. doi: 10.1016/j.clinph.2010.08.021.

Käthner, I., Halder, S., Hintermüller, C., Espinosa, A., Guger, C., Miralles, F., et al. (2017). A multifunctional brain-computer interface intended for home use: An evaluation with healthy participants and potential end users with dry and gel-based electrodes. *Front. Neurosci.* 11, 286. doi: 10.3389/fnins.2017.00286.

Käthner, I., Kübler, A., and Halder, S. (2015). Rapid P300 brain-computer interface communication with a head-mounted display. *Front. Neurosci.* 9, 207. doi: 10.3389/fnins.2015.00207.

Kaufmann, T., Holz, E. M., and Kübler, A. (2013a). Comparison of tactile, auditory, and visual modality for brain-computer interface use: A case study with a patient in the locked-in state. *Front. Neurosci.* 7, 129. doi: 10.3389/fnins.2013.00129.

Kaufmann, T., Schulz, S. M., Koblitz, A., Renner, G., Wessig, C., and Kübler, A. (2013b). Face stimuli effectively prevent brain-computer interface inefficiency in patients with neurodegenerative disease. *Clin. Neurophysiol.* 124, 893–900. doi: 10.1016/j.clinph.2012.11.006.

Kleih, S. C., Gottschal, L., Teichlein, E., and Weilbach, F. X. (2016). Toward a P300 based brain-computer interface for aphasia rehabilitation after stroke: Presentation of theoretical considerations and a pilot feasibility study. *Front. Hum. Neurosci.* 10, 547. doi: 10.3389/fnhum.2016.00547.

Kleih, S. C., Herweg, A., Kaufmann, T., Staiger-Sälzer, P., Gerstner, N., and Kübler, A. (2015). The WIN-speller: A new intuitive auditory brain-computer interface spelling application. *Front. Neurosci.* 9, 346. doi: 10.3389/fnins.2015.00346.

Krausz, G., Ortner, R., and Opisso, E. (2011). Accuracy of a Brain Computer Interface (P300 spelling device) used by people with motor impairments. *Studies in health technology and informatics* 167, 182–186. doi: 10.3233/978-1-60750-766-6-182

Kübler, A., Furdea, A., Halder, S., Hammer, E. M., Nijboer, F., and Kotchoubey, B. (2009). A brain-computer interface controlled auditory event-related potential (p300) spelling system for locked-in patients. *Ann. N.Y. Acad. Sci.* 1157, 90–100. doi: 10.1111/j.1749-6632.2008.04122.x.

Kübler, A., Kotchoubey, B., Hinterberger, T., Ghanayim, N., Perelmouter, J., Schauer, M., et al. (1999). The thought translation device: a neurophysiological approach to communication in total motor paralysis. *Exp. Brain Res.* 124, 223–32. doi: 10.1007/s002210050617.

Leeb, R., Perdikis, S., Tonin, L., Biasiucci, A., Tavella, M., Creatura, M., et al. (2013). Transferring brain-computer interfaces beyond the laboratory: Successful application control for motor-disabled users. *Artif. Intell. Med.* 59, 121–132. doi: 10.1016/j.artmed.2013.08.004.

Lesenfants, D., Habbal, D., Lugo, Z., Lebeau, M., Horki, P., Amico, E., et al. (2014). An independent SSVEP-based brain-computer interface in locked-in syndrome. *J. Neural Eng.* 11, 035002. doi: 10.1088/1741-2560/11/3/035002.

Lugo, Z. R., Rodriguez, J., Lechner, A., Ortner, R., Gantner, I. S., Laureys, S., et al. (2014). A vibrotactile P300-based brain-computer interface for consciousness detection and communication. *Clin. EEG and Neurosci.* 45, 14–21. doi: 10.1177/1550059413505533.

Lulé, D., Noirhomme, Q., Kleih, S. C., Chatelle, C., Halder, S., Demertzi, A., et al. (2013). Probing command following in patients with disorders of consciousness using a brain-computer interface. *Clin. Neurophysiol.* 124, 101–106. doi: 10.1016/j.clinph.2012.04.030.

Mainsah, B. O., Collins, L. M., Colwell, K. A., Sellers, E. W., Ryan, D. B., Caves, K., et al. (2015). Increasing BCI communication rates with dynamic stopping towards more practical use: An ALS study. *J. Neural Eng.* 12:016013. doi: 10.1088/1741-2560/12/1/016013.

Mak, J. N., McFarland, D. J., Vaughan, T. M., McCane, L. M., Tsui, P. Z., Zeitlin, D. J., et al. (2012). EEG correlates of P300-based brain-computer interface (BCI) performance in people with amyotrophic lateral sclerosis. *J. Neural Eng.* 9:026014. doi: 10.1088/1741-2560/9/2/026014.

Mangia, A. L., Pirini, M., Simoncini, L., and Cappello, A. (2014). A feasibility study of an improved procedure for using EEG to detect brain responses to imagery instruction in patients with disorders of consciousness. *PLoS ONE* 9, e99289. doi: 10.1371/journal.pone.0099289.

Marchetti, M., Piccione, F., Silvoni, S., Gamberini, L., and Priftis, K. (2013). Covert visuospatial attention orienting in a brain-computer interface for amyotrophic lateral sclerosis patients. *Neurorehabil. Neural Repair* 27, 430–438. doi: 10.1177/1545968312471903.

McCane, L. M., Heckman, S. M., McFarland, D. J., Townsend, G., Mak, J. N., Sellers, E. W., et al. (2015). P300-based brain-computer interface (BCI) event-related potentials (ERPs): People with amyotrophic lateral sclerosis (ALS) vs. age-matched controls. *Clin. Neurophysiol.* 126, 2124–2131. doi: 10.1016/j.clinph.2015.01.013.

McCane, L. M., Sellers, E. W., McFarland, D. J., Mak, J. N., Carmack, C. S., Zeitlin, D., et al. (2014). Brain-computer interface (BCI) evaluation in people with amyotrophic lateral sclerosis. *Amyotroph. Lateral Scler. Frontotemp. Degen.* 15, 207–215. doi: 10.3109/21678421.2013.865750.

Medina-Juliá, M. T., Fernández-Rodríguez, A., Velasco-Álvarez, F., and Ron-Angevin, R. (2020). P300-Based Brain-Computer Interface Speller: Usability Evaluation of Three Speller Sizes by Severely Motor-Disabled Patients. *Front. Hum. Neurosci.* 14, 583358. doi: 10.3389/fnhum.2020.583358.

Miao, Y., Yin, E., Allison, B. Z., Zhang, Y., Chen, Y., Dong, Y., et al. (2020). An ERP-based BCI with peripheral stimuli: validation with ALS patients. *Cogn. Neurodyn.* 14, 21–33. doi: 10.1007/s11571-019-09541-0.

Naito, M., Michioka, Y., Ozawa, K., Ito, Y., Kiguchi, M., and Kanazawa, T. (2007). A communication means for totally locked-in ALS patients based on changes in cerebral blood volume measured with near-infrared light. *IEICE Trans. Inform. Syst.* E90-D, 1028–1037. doi: 10.1093/ietisy/e90-d.7.1028.

Nam, C. S., Woo, J., and Bahn, S. (2012). Severe motor disability affects functional cortical integration in the context of brain-computer interface (BCI) use. *Ergonomics* 55, 581–591. doi: 10.1080/00140139.2011.647095.

Neshige, R., Murayama, N., Igasaki, T., Tanoue, K., Kurokawa, H., and Asayama, S. (2007). Communication aid device utilizing event-related potentials for patients with severe motor impairment. *Brain Res.* 1141, 218–227. doi: 10.1016/j.brainres.2006.12.003.

Neumann, N., Kübler, A., Kaiser, J., Hinterberger, T., and Birbaumer, N. (2003). Conscious perception of brain states: mental strategies for brain-computer communication. *Neuropsychologia* 41, 1028–36. doi: 10.1016/S0028-3932(02)00298-1.

Neuper, C., Müller, G. R., Kübler, A., Birbaumer, N., and Pfurtscheller, G. (2003). Clinical application of an EEG-based brain-computer interface: a case study in a patient with severe motor impairment. *Clin. Neurophysiol.* 114, 399–409. doi: 10.1016/S1388-2457(02)00387-5.

Nijboer, F., Sellers, E. W., Mellinger, J., Jordan, M. A., Matuz, T., Furdea, A., et al. (2008). A P300-based brain-computer interface for people with amyotrophic lateral sclerosis. *Clin. Neurophysiol.* 119, 1909–16. doi: 10.1016/j.clinph.2008.03.034.

Oken, B. S., Orhan, U., Roark, B., Erdogmus, D., Fowler, A., Mooney, A., et al. (2014). Brain-computer interface with language model-electroencephalography fusion for locked-in syndrome. *Neurorehabil. Neural Repair* 28, 387–394. doi: 10.1177/1545968313516867.

Onishi, A., Takano, K., Kawase, T., Ora, H., and Kansaku, K. (2017). Affective stimuli for an auditory P300 brain-computer interface. *Front. Neurosci.* 11, 522. doi: 10.3389/fnins.2017.00522.

Ortner, R., Aloise, F., Pruckl, R., Schettini, F., Putz, V., Scharinger, J., et al. (2011). Accuracy of a P300 speller for people with motor impairments: a comparison. *Clin. EEG Neurosci.* 42, 214–8. doi: 10.1177/155005941104200405

Pandarinath, C., Nuyujukian, P., Blabe, C. H., Sorice, B. L., Saab, J., Willett, F. R., et al. (2017). High performance communication by people with paralysis using an intracortical brain-computer interface. *eLife* 6, e18554. doi: 10.7554/eLife.18554.

Perdikis, S., Leeb, R., Williamson, J., Ramsay, A., Tavella, M., Desideri, L., et al. (2014). Clinical evaluation of BrainTree, a motor imagery hybrid BCI speller. *J. Neural Eng.* 11, 036003. doi: 10.1088/1741-2560/11/3/036003.

Peters, B., Bedrick, S., Dudy, S., Eddy, B., Higger, M., Kinsella, M., et al. (2020). SSVEP BCI and Eye Tracking Use by Individuals With Late-Stage ALS and Visual Impairments. *Front. Hum. Neurosci.* 14, 595890. doi: 10.3389/fnhum.2020.595890.

Pires, G., Nunes, U., and Castelo-Branco, M. (2011). Statistical spatial filtering for a P300-based BCI: Tests in able-bodied, and patients with cerebral palsy and amyotrophic lateral sclerosis. *J. Neurosci. Methods* 195, 270–281. doi: 10.1016/j.jneumeth.2010.11.016.

Pires, G., Nunes, U., and Castelo-Branco, M. (2012). Comparison of a row-column speller vs. a novel lateral single-character speller: Assessment of BCI for severe motor disabled patients. *Clin. Neurophysiol.* 123, 1168–1181. doi: 10.1016/j.clinph.2011.10.040.

Riccio, A., Holz, E. M., Arico, P., Leotta, F., Aloise, F., Desideri, L., et al. (2015). Hybrid P300-based brain-computer interface to improve usability for people with severe motor disability: electromyographic signals for error correction during a spelling task. *Arch. Phys. Med. Rehabil.* 96 (3 Suppl), S54-61. doi: 10.1016/j.apmr.2014.05.029.

Riccio, A., Simione, L., Schettini, F., Pizzimenti, A., Inghilleri, M., Belardinelli, M. O., et al. (2013). Attention and P300-based BCI performance in people with amyotrophic lateral sclerosis. *Front. Hum. Neurosci.*7, 732. doi: 10.3389/fnhum.2013.00732.

Ron-Angevin, R., Varona-Moya, S., and da Silva-Sauer, L. (2015). Initial test of a T9-like P300-based speller by an ALS patient. *J. Neural Eng.* 12, 046023. doi: 10.1088/1741-2560/12/4/046023.

Scherer, R., Billinger, M., Wagner, J., Schwarz, A., Hettich, D. T., Bolinger, E., et al. (2015). Thought-based row-column scanning communication board for individuals with cerebral palsy. *Ann. Phys. Rehabil. Med.* 58, 14–22. doi: 10.1016/j.rehab.2014.11.005.

Schettini, F., Riccio, A., Simione, L., Liberati, G., Caruso, M., Frasca, V., et al. (2015). Assistive device with conventional, alternative, and brain-computer interface inputs to enhance interaction with the environment for people with amyotrophic lateral sclerosis: A feasibility and usability study. *Arch. Phys. Med. Rehabil.* 96, S46–S53. doi: 10.1016/j.apmr.2014.05.027.

Schreuder, M., Riccio, A., Risetti, M., Dahne, S., Ramsay, A., Williamson, J., et al. (2013). User-centered design in brain-computer interfaces-a case study. *Artif. Intell. Med.* 59, 71–80. doi: 10.1016/j.artmed.2013.07.005.

Schudlo, L. C., and Chau, T. (2018). Development and testing an online near-infrared spectroscopy brain-computer interface tailored to an individual with severe congenital motor impairments. *Disabil. Rehabil. Assist. Technol.* 13, 581–591. doi: 10.1080/17483107.2017.1357212.

Sellers, E. W., Krusienski, D. J., McFarland, D. J., Vaughan, T. M., and Wolpaw, J. R. (2006). A P300 event-related potential brain-computer interface (BCI): The effects of matrix size and inter stimulus interval on performance. *Biol. Psychology* 73, 242–252. doi: 10.1016/j.biopsycho.2006.04.007.

Sellers, E. W., Ryan, D. B., and Hauser, C. K. (2014). Noninvasive brain-computer interface enables communication after brainstem stroke. *Sci. Transl. Med.* 6, 257re7. doi: 10.1126/scitranslmed.3007801.

Sellers, E. W., Vaughan, T. M., and Wolpaw, J. R. (2010). A brain-computer interface for long-term independent home use. *Amyotroph. Lateral Scler.* 11, 449–455. doi: 10.3109/17482961003777470.

Severens, M., Van der Waal, M., Farquhar, J., and Desain, P. (2014). Comparing tactile and visual gaze-independent brain-computer interfaces in patients with amyotrophic lateral sclerosis and healthy users. *Clin. Neurophysiol.* 125, 2297–2304. doi: 10.1016/j.clinph.2014.03.005.

Silvoni, S., Cavinato, M., Volpato, C., Ruf, C. A., Birbaumer, N., and Piccione, F. (2013). Amyotrophic lateral sclerosis progression and stability of brain-computer interface communication. *Amyotroph. Lateral Scler. Frontotemp. Degen.* 14, 390–396. doi: 10.3109/21678421.2013.770029.

Simon, N., Käthner, I., Ruf, C. A., Pasqualotto, E., Kübler, A., and Halder, S. (2015). An auditory multiclass brain-computer interface with natural stimuli: Usability evaluation with healthy participants and a motor impaired end user. *Front. Hum. Neurosci.* 8, 1039. doi: 10.3389/fnhum.2014.01039.

Spuler, M., Bensch, M., Kleih, S., Rosenstiel, W., Bogdan, M., and Kübler, A. (2012). Online use of error-related potentials in healthy users and people with severe motor impairment increases performance of a P300-BCI. *Clin. Neurophysiol.* 123, 1328–37. doi: 10.1016/j.clinph.2011.11.082.

Townsend, G., LaPallo, B. K., Boulay, C. B., Krusienski, D. J., Frye, G. E., Hauser, C. K., et al. (2010). A novel P300-based brain-computer interface stimulus presentation paradigm: Moving beyond rows and columns. *Clin. Neurophysiol.* 121, 1109–1120. doi: 10.1016/j.clinph.2010.01.030.

Vansteensel, M. J., Pels, E. G. M., Bleichner, M. G., Branco, M. P., Denison, T., Freudenburg, Z. V., et al. (2016). Fully Implanted Brain-Computer Interface in a Locked-In Patient with ALS. *N. Engl. J. Med.* 375, 2060–2066. doi: 10.1056/NEJMoa1608085.

Wang, F., He, Y., Qu, J., Xie, Q., Lin, Q., Ni, X., et al. (2017). Enhancing clinical communication assessments using an audiovisual BCI for patients with disorders of consciousness. *J. Neural Eng.* 14, 046024. doi: 10.1088/1741-2552/aa6c31.

Wolpaw, J. R., Bedlack, R. S., Reda, D. J., Ringer, R. J., Banks, P. G., Vaughan, T. M., et al. (2018). Independent home use of a brain-computer interface by people with amyotrophic lateral sclerosis. *Neurology* 91, e258–e267. doi: 10.1212/WNL.0000000000005812.

Zickler, C., Halder, S., Kleih, S. C., Herbert, C., and Kübler, A. (2013). Brain painting: Usability testing according to the user-centered design in end users with severe motor paralysis. *Artif. Intell. Med.* 59, 99–110. doi: 10.1016/j.artmed.2013.08.003.

Zickler, C., Riccio, A., Leotta, F., Hillian-Tress, S., Halder, S., Holz, E., et al. (2011). A brain-computer interface as input channel for a standard assistive technology software. *Clin. EEG Neurosci.* 42, 236–44. doi: 10.1177/155005941104200409.
