# Supplementary material for: A systematic review of research on augmentative and alternative communication brain-computer interface systems for individuals with disabilities
Source: Front Hum Neurosci. 2022 Jul 27;16:952380. doi: 10.3389/fnhum.2022.952380 (PMC9374067; doi:10.3389/fnhum.2022.952380)
Supplement: Supplementary file 2 [file Table_2.docx]

**Supplementary table 2.** Summary of key study, system, and participant characteristics, and communication task performance, for studies including participants both with and without disabilities. See supplementary table 1 for additional details on each study listed here. For studies reporting accuracy for a smaller sample than that indicated in the sample size column (i.e., due to participant exclusion), the sample size for accuracy calculation is indicated. Statistical comparisons between participants with disabilities and control participants without disabilities are summarized, when applicable. “ERP” refers to P300 and N200 signals. Studies are organized first by interface type and then in alphabetical order. For studies reporting accuracy for a smaller sample than that indicated in the sample size column (i.e., due to participant exclusion), the sample size for accuracy calculation is indicated. PWD=participants with disabilities. Con=control participants without disabilities. N/R=not reported. M=mean. SD=standard deviation. R=range. SEM=standard error of the mean. Med=median. Dx=Diagnosis. n=sample size. Standard medical abbreviations were provided for medical diagnoses. These included: ALS=amyotrophic lateral sclerosis. OPCA=olivopontocerebellar atrophy. CP=cerebral palsy. MD=Muscular Dystrophy. SCI=spinal cord injury. CVA=cerebrovascular accident or stroke. SMA=spinal muscular atrophy. MS=multiple sclerosis. SBMA=spinobulbar muscular atrophy. DOC=disorders of consciousness. S/p=status post. TBI=traumatic brain injury

| **Study** | **Interface type** | **Comm. task** | **n** | | | **PWD dx** | | **Age in years** | | | | **Task differences** | **Accuracy results (%)** | | **Other results** | | **Statistical comparison** |
| --- | --- | --- | --- | --- | --- | --- | --- | --- | --- | --- | --- | --- | --- | --- | --- | --- | --- |
|  |  |  | **PWD** | **Con** | |  |  | **PWD** | **Con** | | |  | **PWD** | **Con** | **PWD** | **Con** |  |
| ***EEG, ERP, Visual*** | | | | | | | | | | | | | | | | | |
| Clements et al., 2016 | Matrix speller | Copy-spell  (word) | 8 | 26 | ALS (n=7), PLS (n=1) | | N/R | | | N/R | | Some Con did not use dynamic stopping (n=16) | *Wet*  M=87.9  R=67-100  *Dry*  M=50.9  R=3-90 | With dynamic stopping algorithm (n=10):  *Wet*  M=94.3  *Dry*  M=79.0 | Bit rate (bits/min):  *Wet*  M=32.5  *Dry*  M=8.9 | Bit rate (bits/min):  *Wet*  M=38.7  *Dry*  M=17.9 | N/R |
| Cruz, Pires, & Nunes, 2018 | Lateral single character speller | Copy-spell (sentence) | 1 | 9 | SCI | | N/R | | | 24-43 | | None | *With EC* M=79.6; *Without EC*  M=75.0 | *With EC*: M=91.3 SD=5.77 *Without EC*  M=86.2 SD=8.12 | ITR (bits/min):  *With EC*  M=9.5 *Without EC*  M=8.6 | ITR (bits/min):  *With EC*  M=14.9 SD=3.65 *Without EC*  M=13.4 SD=3.40 | N/R |
|  |  |  |  |  |  | |  | | |  | |  |  |  |  |  |  |
| ***EEG, ERP, Visual (continued)*** | | | | | | | | | | | | | | | | | |
| Geronimo, Simmons, & Schiff, 2016 | Matrix speller | Copy-spell  (word) | 25 | 10 | ALS | | Med=58  R=45.5-74 | | | Med=55.5  R=45-73.5 | | 4 sessions for PWD, 2 sessions for Con | M=68  R=0-100 | M=86  R=38-100 | N/R | N/R | Kruskal-Wallis test and post-hoc pairwise comparisons: significantly lower accuracy for PWD with cognitive impairment than for Con, no difference for PWD without cognitive impairment |
| Ikegami et al., 2011 | Matrix speller | Character selection | 10 | 10 | | SCI | | M=41.9 SD=7.55 R=26-53 | | | M=42.1  R=27-52 | None | *Green-blue*  M=90.7  SEM=1.0  *White-gray*  M=88  SEM=1.2 | *Green-blue*  M=86.0 SEM=1.9 *White-gray*  M=77.3 SEM=2.7 | Bit rate (bits/min): *Green-blue*  M=10.2 *White-gray*  M=9.8 | Bit rate (bits/min): *Green-blue*  M=9.6  *White-gray*  M=8.4 | Two-way repeated-measure ANOVA: no main effect for group (PWD vs Con), no significant interaction between group and condition |
| Ikegami et al., 2014 | Matrix speller | Character  selection | 7 | 7 | | ALS | | M=64.1 SD=3.29  R=59-68 | | | M=64.0  R=58-68 | None | *Row-column*  M=23.8  SD=13.1  R=16.7-50  *Two-step* M=54.8  SD=31.5  R=16.7-100 | *Row-column*  M=71.4 SD=32.9 R=16.7-100  *Two-step* M=83.3 SD=9.6 R=66.7-100 | N/R | N/R | Kruskal-Wallis test: significant difference between PWD and Con under row-column condition but not two-step condition |
|  |  |  |  |  | |  | |  | | |  |  |  |  |  |  |  |
| ***EEG, ERP, Visual (continued)*** | | | | | | | | | | | | | | | | | |
| Käthner, Kübler, & Halder, 2015 | Matrix speller | Copy-spell (word) | 1 | 18 | | ALS | | 80 | | | M=25 SD=3.9  R=21-34 | Con tested 2 VR headset conditions and 22" monitor condition, PWD tested 1 VR condition, 22" monitor, and 32" monitor | *TV display* M=33  *22" monitor*  M=50.3  R=18-67  *VR*  M=45.3  R=17-100 | *22" monitor* M=94 SD=5.3  *VR*  M=94 SD=8.8 | N/R | Bit rate (bits/min):  *VR headset*  M=15.5  *22" monitor* M=15.5 | N/R |
| Kaufmann et al., 2013 | Matrix speller | Copy-spell  (word) | 9 | 16 | | ALS (n=4), MD (n=1), SMA (n=2), SBMA (n=2) | | M=50.0 SD=15.21 R=25-72 | | | M=23.7 SD=2.6 R=19-33 | 3 conditions for PWD, 4 for Con. | With 1 sequence:  *Famous FF*  M=85.7  R=40-100  *Personal FF*  M=77.1  R=20-100  *CF*  M=11.4  R=0-40 | With 1 sequence:  *Famous FF*  M=~88  *Personal FF*  M=~78  *CF*  M=~43 | Bit rate with 1 sequence (bits/min):  *Famous FF*  M=52  *Personal FF*  M=45  *CF*  M=3 | N/R | Mann-Whitney U test: significantly lower accu-racy for PWD than for Con with CF, no significant difference with famous or personal FF |
| McCane et al., 2015 | Matrix speller | Copy-spell  (word) | 14 | 14 | | ALS | | M=55.9 SD=9.4  R=41-72 | | | M=55.8 SD=9.0 R=39-69 | None | M=95.7  SE=2 | M=98.8  SE=1 | Characters per minute (cpm):  M=2.1  SEM=0.3  Bit rate (bit/min)  M=11.2  SE=1.3 | Characters per minute (cpm):  M=2.6  SE=0.2  Bit rate (bits/min):  M=13.7  SE=1.1 | Mann-Whitney U test: no significant difference between PWD and Con for accuracy;  T-test: no significant difference between PWD and Con for characters/min or bits/min |
| Nam, Woo, & Bahn, 2012 | Matrix speller | Copy-spell  (phrase) | 9 | 9 | | ALS (n=3),  CP (n=6) | | M=41.2 SD=11.3 | | | M=25.3 SD=2.3 | None | M=36  SD=3.3 | M=88  SD=6 | ITR (bits/min)  M=0.7  SD=0.91 | ITR (bits/min)  M=2.0  SD=0.40 | T-test; significantly lower accuracy and ITR for PWD than for Con |
| ***EEG, ERP, Visual (continued)*** | | | | | | | | | | | | | | | | | |
| Neshige et al., 2007 | 4-choice words and hiragana matrix speller | Multiple-choice response selection (field:4), character selection, icon selection (HC only) | 6 | 17 | | ALS (n=5), OPCA(n=1) | | M=60.5 SD=12.10 R=48-80 | | | M=25 SD=9 years | None | *Word (n=5)*  M=100  *Character (n=3)*  M=100 | *Word*  M=100  *Character* M=87 | N/R | N/R | N/R |
| Oken et al., 2014 | Rapid serial visual presentation (RSVP) | Copy-spell (word) with levels of difficulty | 9 | 9 | | ALS(n=5),  MD(n=1) | | M=45.8  R=27-65 | | | M=45.2  R=17-66 | None | *L1 (n=6)*  6/6 complete M=90.9  R=33.3-100  *L2 (n=6)*  4/6 complete M=90.7 R=28.6-100  *L3 (n=4)*  2/4 complete  M=95.9  R=75.0-100  *L4 (n=2)*  1/2 complete M=100 R=100-100  *L5 (n=1)*  1/1 complete M=84.6 R=42.9-100 | *L1 (n=9)*  9/9 complete M=94.1 R=28.6-100  *L2 (n=9)*  8/9 complete M=89.8  R=0-100  *L3 (n=8)*  7/8 complete M=93.3 R=66.7-100  *L4 (n=7)*  6/7 complete M=79.0 R=28.6-100  *L5 (n=6)*  6/6 complete M=88.7, R=66.7-100 | Correct characters per minute (ccpm):  *L1* (n=6)  M=1.8  R=0.9-3.7  *L2* (n=6)  M=1.5  R=0.0-2.5  *L3* (n=4)  M=0.4  R=0.0-0.9  *L4* (n=2)  M=3.3  R=3.3-3.3  *L5* (n=1)  M=2.3  R=1.6-3.0 | Correct characters per minute (ccpm)  *L1 (n=9)* M=2.5  R=1.0-3.5  *L2 (n=9)* M=2.3  R=0.6-3.1  *L3 (n=8)* M=1.5  R=0.2-2.6  *L4 (n=7)* M=1.2  R=0.3-2.  *L5 (n=6)* M=1.6  R=0.9-2.7 | Mann-Whitney U test: difference in highest difficulty level completed approached significance |
| Pires, Nunes, Castelo-Branco, 2011 | Matrix speller | Copy-spell  (sentence) | 5 | 19 | | ALS (n=2), CP (n=3) | | M=48.0 SD=23.4 R=18-75 | | | M=30.1  R=18-42 | Different target sentences | CP  M=96.7  ALS  M=96.9 | Group 1 (longer sentence) M=91.0 Group 2 (shorter sentence) M=90.32 | Symbols/min (spm):  CP  M=3.1  ALS  M=3.8  Bandwidth (bits/min):  CP  M=15.1  ALS  M=18.2 | Symbols/min (spm)  Group 1  M=4.3  Group 2  M=4.9  Bandwidth:  (bits/min)  Group 1  M=18.8  Group 2  M=21.3 | N/R |
|  |  |  |  |  | |  | |  | | |  |  |  |  |  |  |  |
| ***EEG, ERP, Visual (continued)*** | | | | | | | | | | | | | | | | | |
| Pires, Nunes, Castelo-Branco, 2012 | *Matrix speller & lateral single character speller (LSC)* | Copy-spell  (sentence) | 14 | 10 | | ALS (n=7),  MD (n=1),  CP (n=5),  SCI (n=1) | | M=53.2 SD=21.17 R=18-80 | | | M=29.2,  R=24-38 | Different target sentences | *Matrix*  ALS (n=6)  M=91.7  Other Dx (n=4)  M=85.4  *LSC*  ALS (n=5)  M=91.3  Other Dx (n=3)  M=81.6 | *Matrix (n=9)*  M=89.3  *LSC*  M=97.7 | Symbols per minute (spm):  *Matrix*  ALS  M=4.1  Other Dx  M=3.3  *LSC*  ALS  M=5.0  Other Dx  M=3.5  ITR (bits/min):  *Matrix*  ALS  M=17.8  Other Dx  M=13.0  *LSC*  ALS  M=20.0  Other Dx  M=11.6 | Symbols per minute (spm): *Matrix (n=9)*  M=4.5  *LSC*  M=5.5  ITR (bits/min)  *Matrix*  (n=9)  M=18.9  *LSC*  (n=10)  M=22.2 | N/R |
| Riccio et al., 2015 | Matrix speller | Copy-spell  (word) | 3 | 8 | | ALS (n=1),  CVA (n=2) | | M=50.3 SD=3.2 R=48-54 | | | M=28 SD=7 | None | *With EC*  M=97.3  R=92-100, *Without EC*  M=73.7  R=63-93 | *With EC* Med=100 R=84.5-100  *Without EC* Med=70.3  R=52-84.5 | ITR (bits/min):  *With EC*  Med=7.2  R=5.9-12.5 *Without EC*  Med=4.1  R=1.5-5.0 | ITR (bits/min):  *With EC*  Med=12  R=1.8-13.3 *Without EC* Med=4.7  R=0.6-8.9 | N/R |
| Ron-Angevin, Varona-Moya, & da Silva-Sauer, 2015 | *Matrix speller & T9 speller* | Copy-spell (sentence) | 1 | 11 | | ALS | | 62 | | | M=26.2 SD=4.64 R=19-36 | Con tested T9 and matrix with and without word prediction, PWD tested only T9 and matrix with word prediction | *Matrix*  93.3  *T9*  88.2 | *Matrix*  M=91.9 SD=8.92  *T9*  M=91.7  SD=10.09 | Time to copy-spell 7-word sentence (s):  *Matrix*  1035  *T9*  663 | Time to copy-spell 7-word sentence (s):  *Matrix*  Med=931.5  *T9*  Med=585 sec | N/R |
|  | | | | | | | | | | | | | | | | | |
| ***EEG, ERP, Visual (continued)*** | | | | | | | | | | | | | | | | | |
| Spuler  et al.,  2012 | Matrix speller | Copy-spell (target unspecified) | 6 | 17 | | ALS (n=5),  MD (n=1) | | M=51.2 SD=10.2 R=36-63 | | | Young adults (n=9): M=24.6 SD=2.3  R=20-28 Age-match (n=8): M=45.0 SD=5.2 R=39-52 | None | N/R | N/R | Bit rate  (bits/min):  *With error correction*  M=2.1  SD=1.07 | Bit rate:  (bits/min):  *Young adults*  M=2.7  SD=0.71  *Age-match*  M=2.1  SD=1.23 | N/R |
| Townsend  et al., 2010 | Matrix speller | Copy-spell (word & number) | 3 | 18 | | ALS | | N/R | | | N/R | Different stimulus onset asynchrony, time between selections, and number of character selections | *CBP*  M=84  R=78-89  *RCP*  M=59  R=46-68 | *CBP*  M=91.5 SD=5.38  *RCP*  M=77.3 SD=4.48 | Practical bit rate (bits/min):  *CBP*  M=8.9  SD=5.19  R=3.3-13.5 | Practical bit rate  (bits/min)  *CBP*  M=22.6  SD=6.542  R=12.7-39.4 | N/R |
| ***EEG, ERP, Auditory*** | | | | | | | | | | | | | | | | | |
| Halder et al., 2016b | Auditory with visual aid and spatial cues, syllables for selection of consonant and vowel in hiragana | Character selection | 1 | 6 | | SCI | | 43 | | | M=32.6 | None | *Session 1*  12  *Session 2*  28  *Session 3*  56 | *Session 1*  M=43  SD=29  R=0-80  *Session 2*  M=53  SD=36  R=0-88  *Session 3*  M=57  SD=39  R=7-92 | ITR (bits/min):  *Session 1*  0.2  *Session 2*  0.7  *Session 3*  2.0 | ITR (bits/min):  *Session 1*  M=1.8  SD=1.6  R=0-4.2 *Session 2*  M=2.7  SD=2.2  R=0-5.5  *Session 3*  M=3.3  SD=2.7  R=0.1-6.2 | N/R |
| Hill et al., 2014 | Auditory, words | Yes/no questions | 2 | 14 | | ALS | | M=58.5 | | | M=39 SD=17.8 R=22-67 | Con did oddball tasks with words and beeps, PWD did yes/no task with words only | M=84.4 | *Day 1* M=76.9 SD=11.1  *Day 2* M=70.2 SD=11.9 | N/R | N/R | N/R |
|  | | | | | | | | | | | | | | | | | |
| ***EEG, ERP, Auditory (continued)*** | | | | | | | | | | | | | | | | | |
| Kleih et al., 2015 | Auditory, words containing target letters | Copy-spell  (word) | 4 | 11 | | ALS (n=1),  MD (n=1), traumatic accident (n=2) | | M=55.5 SD=12.6 R=43-72 | | | M=23.6 SD=3.6 | Con tested auditory, visual, and multimodal paradigms, PWD tested auditory only | M=74.2  SD=15.0 R=51.9-84.2 | M=83.7 SD=20.73 | ITR (bits/min):  M=1.3  SD=0.8 | ITR (bits/min):  M=1.1  SD=0.7 | N/R |
| Lulé et al., 2013 | Auditory, words | Yes/no questions | 18 | 16 | | CVA(n=2),  DOC s/p anoxic injury (n=2),  DOC s/p  TBI (n=5), DOC unspecified etiology (n=9) | | LIS (n=2)  R=29-63 MCS (n=13) M=42 SD=21 UWS (n=3)  M=61 SD=17 | | | Younger (n=10) M=29 SD=6 Older  (n=6) M=66 SD=7 | 10 questions for PWD, 12 for Con | LIS  M=40.0  SD=28.3  R=20-60  MCS  M=26.8 SD=14.1  R=0-50  UWS  M=33.3 SD=5.8  R=30-40 | Younger M=73.1  SD=23.5 Older M=73.6  SD=23.8 | N/R | N/R | N/R |
| Onishi et al., 2017 | Auditory, positive and negative affective sounds | Yes/no questions | 1 | 15 | | ALS | | 61 | | | M=29 SD=7.2 | Con tested permuted and non-permuted affective stimuli, PWD tested non-permuted only | 90 | *Positive affective*  M=84.7  R=60-100  *Negative affective*  M=77.3  R=50-80 | N/R | N/R | N/R |
| Simon et al., 2015 | Auditory with visual aid, animal sounds | Copy-spell (word) | 1 | 11 | | ALS | | 66 | | | M=24.3 SD=7.1 | 2 sessions over 2 days for Con,  1 month apart for PWD | *Session 1*  M=20  SD=9.7  *Session 2*  M=47  SD=20.1 | *Session 1* M=76.7 SD=21.6 *Session 2* M=90.2 SD=9.3 | Bit rate: (bits/min): *Session 1* M=0.3  *Session 2* M=1.4 | Bit rate:  (bits/min):  *Session 1*  M=3.3  SD=1.30  *Session 2*  M=4.2  SD=0.81 | N/R |
|  | | | | | | | | | | | | | | | | | |
| ***EEG, ERP, Visual & auditory & audiovisual*** | | | | | | | | | | | | | | | | | |
| Sellers et al., 2006 | *Visual, 4-choice words, auditory, 4-choice words, & audio-visual (AV), 4-choice words* | Yes/no questions, binary-choice response selection | 3 | 3 | | ALS | | M=43.7 SD=6.5  R=37-50 | | | M=33.7 SD=3.1 R=31-37 | None | *Visual*  M=63.4  SD=10.0  R=53.9-73.8 *Auditory*  M=65.9  SD=7.1  R=59.1-73.2  *AV*  M=65.1  SD=3.7  R=61.6-64.9 | *Visual* M=68.6 SD=16.9 R=49.3-80.4 *Auditory* M=64.0  SD=6.8 R=56.8-70.2 *AV*  M=71.0  SD=6.8 R=64.6-78.1 | N/R | N/R | Mixed design factorial ANOVA: no main effect for group (PWD vs Con) |
| ***EEG, ERP, visual & tactile*** | | | | | | | | | | | | | | | | | |
| Severens et al., 2014 | *Visual, multi-step speller & tactile with visual aid, 5-tactor multi-step speller* | Copy-spelling (target unspecified) | 5 | 6 | | ALS | | M=39.0 SD=13.8 R=23-56 | | | M=20.0  SD=0.4 | None | *Tactile*  M=53  *Visual*  M=85 | *Tactile*  M=56  *Visual*  M=87 | ITR (bits/min):  *Tactile*  M=6.6  *Visual*  M=8.7 | ITR  (bits/min)  *Tactile*  M=6.6  *Visual*  M=12.1 | N/R |
| ***EEG, ERP & MI, Tactile (ERP) & task instructions only (MI)*** | | | | | | | | | | | | | | | | | |
| Guger et al., 2017 | *Tactile, 3 tactors (ERP) & task instructions only (MI)* | Yes/no questions | 12 | 3 | | ALS | | M=62.2 SD=11.2 R=37-76 | | | M=41.0 SD=2.7 R=38-43 | Con did 2 communication runs with each modality, some PWD tried only one modality | *Tactile (n=9)*  M=80.0 SD=8.7  R=70-90;  *MI (n=3)*  M=93.3 | *Tactile run 1*  M=73.3  SD=46.2 R=20-100  *Tactile run 2*  M=86.7 SD=11.6 R=80-100  *MI run 1* M=73.3 SD=11.6 R=60-80  *MI run 2* M=83.3 SD=5.8  R=80-90 | N/R | N/R | N/R |
|  | | | | | | | | | | | | | | | | | |
| ***EEG, MI*** | | | | | | | | | | | | | | | | | |
| Mangia et al., 2014 | Task instructions only, two mental states used for binary-choice responses | Yes/no questions | 5 | 5 | | DOC s/p TBI | | M=42.0 SD=18.5 R=22-63 | | | R=26-37 | None | M=91.7 SD=7.4 | M=80.7 SD=11.2 | N/R | N/R | N/R |
| Perdikis et al., 2014 | Visual, multi-step speller | Copy-spell  (word) | 6 | 10 | | MD (n=1), SCI (n=5) | | M=39.0 SD=15.5 R=19-60 | | | M=29.0 SD=4.6 | None | Word completion success rate:  *hBCI+CA (n=6)*  100  *hBCI (n=6)*  95.8  *CA (n=2)*  100 | Word completion success rate:  *hBCI+CA* M=100  *hBCI*  M=97.5  *CA*  M=77.8 | For all participants (not reported separately for PWD):  Seconds per character (spc):  *hBCI+CA*  M=35.7  *hBCI*  M=44.9  *CA*  M=34.2  Characters per minute (cpm):  *hBCI+CA*  M=1.7  *hBCI*  M=1.3  *CA*  M=1.8 | | N/R |
| ***EEG, SSVEP, Visual*** | | | | | | | | | | | | | | | | | |
| Lesenfants et al., 2014 | Visual, binary choice color stimuli | Yes/no questions | 6 | 12 | | CVA (n=5), TBI (n=1) | | M=49.0 SD=19.7  R=23-74 | | | M=24.1 SD=3.0 R=21-30 | None | (n=4): M=59.5  SD=9.0 | M=74.0 SD=12.5 | N/R | N/R | N/R |

**References**

Clements, J. M., Sellers, E. W., Ryan, D. B., Caves, K., Collins, L. M., and Throckmorton, C. S. (2016). Applying dynamic data collection to improve dry electrode system performance for a P300-based brain-computer interface. *J. Neural Eng.* 13:066018. doi: 10.1088/1741-2560/13/6/066018.

Cruz, A., Pires, G., and Nunes, U. J. (2018). Double ErrP Detection for Automatic Error Correction in an ERP-Based BCI Speller. *IEEE Trans. Neural Syst. Rehabil. Eng.* 26, 26–36. doi: 10.1109/TNSRE.2017.2755018.

Geronimo, A., Simmons, Z., and Schiff, S. J. (2016). Performance predictors of brain-computer interfaces in patients with amyotrophic lateral sclerosis. *J. Neural Eng.* 13:026002. doi: 10.1088/1741-2560/13/2/026002.

Guger, C., Spataro, R., Allison, B. Z., Heilinger, A., Ortner, R., Cho, W., et al. (2017). Complete locked-in and locked-in patients: Command following assessment and communication with vibro-tactile P300 and motor imagery brain-computer interface tools. *Front. Neurosci.* 11, 256. doi: 10.3389/fnins.2017.00251.

Halder, S., Takano, K., Ora, H., Onishi, A., Utsumi, K., and Kansaku, K. (2016). An Evaluation of Training with an Auditory P300 Brain-Computer Interface for the Japanese Hiragana Syllabary. Front. Neurosci. 10, 446. doi: 10.3389/fnins.2016.00446.

Hill, N. J., Ricci, E., Haider, S., McCane, L. M., Heckman, S., Wolpaw, J. R., et al. (2014). A practical, intuitive brain-computer interface for communicating “yes” or “no” by listening. *J. Neural Eng.* 11:035003. doi: 10.1088/1741-2560/11/3/035003.

Ikegami, S., Takano, K., Kondo, K., Saeki, N., and Kansaku, K. (2014). A region-based two-step P300-based brain-computer interface for patients with amyotrophic lateral sclerosis. *Clin. Neurophysiol.* 125, 2305–2312. doi: 10.1016/j.clinph.2014.03.013.

Ikegami, S., Takano, K., Saeki, N., and Kansaku, K. (2011). Operation of a P300-based brain-computer interface by individuals with cervical spinal cord injury. *Clin. Neurophysiol.* 122, 991–996. doi: 10.1016/j.clinph.2010.08.021.

Käthner, I., Kübler, A., and Halder, S. (2015). Rapid P300 brain-computer interface communication with a head-mounted display. *Front. Neurosci.* 9, 207. doi: 10.3389/fnins.2015.00207.

Kaufmann, T., Schulz, S. M., Koblitz, A., Renner, G., Wessig, C., and Kübler, A. (2013). Face stimuli effectively prevent brain-computer interface inefficiency in patients with neurodegenerative disease. *Clin. Neurophysiol.* 124, 893–900. doi: 10.1016/j.clinph.2012.11.006.

Kleih, S. C., Herweg, A., Kaufmann, T., Staiger-Sälzer, P., Gerstner, N., and Kübler, A. (2015). The WIN-speller: A new intuitive auditory brain-computer interface spelling application. *Front. Neurosci.* 9, 346. doi: 10.3389/fnins.2015.00346.

Lesenfants, D., Habbal, D., Lugo, Z., Lebeau, M., Horki, P., Amico, E., et al. (2014). An independent SSVEP-based brain-computer interface in locked-in syndrome. *J. Neural Eng.* 11, 035002. doi: 10.1088/1741-2560/11/3/035002.

Lulé, D., Noirhomme, Q., Kleih, S. C., Chatelle, C., Halder, S., Demertzi, A., et al. (2013). Probing command following in patients with disorders of consciousness using a brain-computer interface. *Clin. Neurophysiol.* 124, 101–106. doi: 10.1016/j.clinph.2012.04.030.

Mangia, A. L., Pirini, M., Simoncini, L., and Cappello, A. (2014). A feasibility study of an improved procedure for using EEG to detect brain responses to imagery instruction in patients with disorders of consciousness. *PLoS ONE* 9, e99289. doi: 10.1371/journal.pone.0099289.

McCane, L. M., Heckman, S. M., McFarland, D. J., Townsend, G., Mak, J. N., Sellers, E. W., et al. (2015). P300-based brain-computer interface (BCI) event-related potentials (ERPs): People with amyotrophic lateral sclerosis (ALS) vs. age-matched controls. *Clin. Neurophysiol.* 126, 2124–2131. doi: 10.1016/j.clinph.2015.01.013.

Nam, C. S., Woo, J., and Bahn, S. (2012). Severe motor disability affects functional cortical integration in the context of brain-computer interface (BCI) use. *Ergonomics* 55, 581–591. doi: 10.1080/00140139.2011.647095.

Neshige, R., Murayama, N., Igasaki, T., Tanoue, K., Kurokawa, H., and Asayama, S. (2007). Communication aid device utilizing event-related potentials for patients with severe motor impairment. *Brain Res.* 1141, 218–227. doi: 10.1016/j.brainres.2006.12.003.

Oken, B. S., Orhan, U., Roark, B., Erdogmus, D., Fowler, A., Mooney, A., et al. (2014). Brain-computer interface with language model-electroencephalography fusion for locked-in syndrome. *Neurorehabil. Neural Repair* 28, 387–394. doi: 10.1177/1545968313516867.

Onishi, A., Takano, K., Kawase, T., Ora, H., and Kansaku, K. (2017). Affective stimuli for an auditory P300 brain-computer interface. *Front. Neurosci.* 11, 522. doi: 10.3389/fnins.2017.00522.

Perdikis, S., Leeb, R., Williamson, J., Ramsay, A., Tavella, M., Desideri, L., et al. (2014). Clinical evaluation of BrainTree, a motor imagery hybrid BCI speller. *J. Neural Eng.* 11, 036003. doi: 10.1088/1741-2560/11/3/036003.

Pires, G., Nunes, U., and Castelo-Branco, M. (2011). Statistical spatial filtering for a P300-based BCI: Tests in able-bodied, and patients with cerebral palsy and amyotrophic lateral sclerosis. *J. Neurosci. Methods* 195, 270–281. doi: 10.1016/j.jneumeth.2010.11.016.

Pires, G., Nunes, U., and Castelo-Branco, M. (2012). Comparison of a row-column speller vs. a novel lateral single-character speller: Assessment of BCI for severe motor disabled patients. *Clin. Neurophysiol.* 123, 1168–1181. doi: 10.1016/j.clinph.2011.10.040.

Riccio, A., Holz, E. M., Arico, P., Leotta, F., Aloise, F., Desideri, L., et al. (2015). Hybrid P300-based brain-computer interface to improve usability for people with severe motor disability: electromyographic signals for error correction during a spelling task. *Arch. Phys. Med. Rehabil.* 96 (3 Suppl), S54-61. doi: 10.1016/j.apmr.2014.05.029.

Ron-Angevin, R., Varona-Moya, S., and da Silva-Sauer, L. (2015). Initial test of a T9-like P300-based speller by an ALS patient. *J. Neural Eng.* 12, 046023. doi: 10.1088/1741-2560/12/4/046023.

Sellers, E. W., Krusienski, D. J., McFarland, D. J., Vaughan, T. M., and Wolpaw, J. R. (2006). A P300 event-related potential brain-computer interface (BCI): The effects of matrix size and inter stimulus interval on performance. *Biol. Psychology* 73, 242–252. doi: 10.1016/j.biopsycho.2006.04.007.

Severens, M., Van der Waal, M., Farquhar, J., and Desain, P. (2014). Comparing tactile and visual gaze-independent brain-computer interfaces in patients with amyotrophic lateral sclerosis and healthy users. *Clin. Neurophysiol.* 125, 2297–2304. doi: 10.1016/j.clinph.2014.03.005.

Simon, N., Käthner, I., Ruf, C. A., Pasqualotto, E., Kübler, A., and Halder, S. (2015). An auditory multiclass brain-computer interface with natural stimuli: Usability evaluation with healthy participants and a motor impaired end user. *Front. Hum. Neurosci.* 8, 1039. doi: 10.3389/fnhum.2014.01039.

Spuler, M., Bensch, M., Kleih, S., Rosenstiel, W., Bogdan, M., and Kübler, A. (2012). Online use of error-related potentials in healthy users and people with severe motor impairment increases performance of a P300-BCI. *Clin. Neurophysiol.* 123, 1328–37. doi: 10.1016/j.clinph.2011.11.082.

Townsend, G., LaPallo, B. K., Boulay, C. B., Krusienski, D. J., Frye, G. E., Hauser, C. K., et al. (2010). A novel P300-based brain-computer interface stimulus presentation paradigm: Moving beyond rows and columns. *Clin. Neurophysiol.* 121, 1109–1120. doi: 10.1016/j.clinph.2010.01.030.
