# Supplementary material for: A systematic review of research on augmentative and alternative communication brain-computer interface systems for individuals with disabilities
Source: Front Hum Neurosci. 2022 Jul 27;16:952380. doi: 10.3389/fnhum.2022.952380 (PMC9374067; doi:10.3389/fnhum.2022.952380)
Supplement: Supplementary file 3 [file Data_Sheet_1.docx]

A systematic review of research on augmentative and alternative communication brain-computer interface systems for individuals with disabilities: Supplementary material

# Search terms

**Scopus:**

(TITLE-ABS-KEY("brain computer interface*" OR BCI OR electroencephalography OR eeg OR "evoked potential"OR "event related potential" OR p300 OR "slow OR potential")) AND (TITLE-ABS-KEY("communication aide*" OR communicat* OR conversation* OR converse OR typing OR spell OR spell* OR writing OR write OR articulat* OR "augmentative communication" OR "alternative communication")) AND (adult* OR aged OR elderly OR "middle aged") AND (TITLE-ABS-KEY(human))

**OvidMEDLINE(R):**

1 brain computer interface.mp. or exp Brain-Computer Interfaces/

2 BCI.mp.

3 EEG.mp. or Electroencephalography/

4 Evoked Potentials/

5 Event-Related Potentials.mp.

6 Event-Related Potentials, P300/

7 Evoked Potentials, Visual/

8 Evoked Potentials, Auditory/

9 Evoked Potentials, Motor/

10 Evoked Potentials, Somatosensory/

11 Slow cortical potential.mp.

12 Communication Aids for Disabled/

13 Communication/

14 communicat*.mp.

15 conversation.mp.

16 typing.mp.

17 spell*.mp.

18 articulat*.mp.

19 (writing or write).mp. [mp=title, abstract, original title, name of substance word, subject heading word, keyword heading word, protocol supplementary concept word, rare disease supplementary concept word, unique identifier, synonyms]

20 augmentative communication.mp.

21 alternative communication.mp.

22 12 or 13 or 14 or 15 or 16or 17 or 18 or 19 or 20 or 21

23 1 or 2 or 3 or 4 or 5 or 6 or 7 or 8 or 9 or 10 or 11

24 22 and 23

25 limit 24 to humans

26 adult.mp. or middle aged.sh. or age:.tw.

27 25 and 26

**CINAHL:**

S21 S7 AND S16 AND

S20 S20 S17 OR S18 OR S19

S19 (MH "Aged+") OR (MH "Aged, 80 and Over")

S18 (MH "Middle Age")

S17 (MH "Adult+")

S16 S8 OR S9 OR S10 OR S11 OR S12 OR S13 OR S14 OR S15

S15 (MH "Alternative and Augmentative Communication")

S14 writing OR write OR written

S13 articulat*

S12 spell*

S11 "typing"

S10 convers*

S9 (MH "Communication")

S8 (MH "Communication Aids for Disabled+")

S7 S1 OR S2 OR S3 OR S4OR S5 OR S6

S6 "slow cortical potentials"

S5 (MH "Evoked Potentials+") OR (MH "Evoked Potentials, Visual") OR (MH "Evoked Potentials, Somatosensory") OR (MH "Evoked Potentials, Motor") OR (MH "Evoked Potentials, Auditory+")

S4 EEG

S3 (MH "Electroencephalography")

S2 BCI

S1 (MH "Brain-Computer Interfaces")

# AAC-BCI system and protocol description items

- Were the equipment and manufacturer named?
- Were the number and position of electrodes specified? (e.g., 10-20 position or position using specified cap)
- Was detail provided on how signal artifacts (i.e., eye-blink, movement) were accounted for?
- Was the data acquisition method specified? (e.g., invasive: ECoG, microelectrode; noninvasive: EEG, fMRI, fNIRS, MEG; physiologic: EMG, EOG)
- Was the signal type specified? (e.g., ERP, SSVEP, motor imagery, etc.)
- Were methods of signal processing and analysis adequately described?
- Were consistent instructions provided to all participants? (must indicate use of a script, video, or other consistent method of instruction)
- Was the task adequately described? (e.g., calibration, copy-spelling, free-spelling, binary/multiple choice; number of task repetitions; number of required responses)
- Were stimuli (i.e. what elicits the brain signal) adequately described?
- Was the method of response selection adequately described? (e.g., number of trials per selection, classification method, confidence threshold, language modeling)
- Was the method of stimulus presentation and/or user interface adequately described?
- Did the study describe if feedback was or was not provided to the participant (i.e., closed-loop vs open-loop), and if so, was the method of feedback adequately described (modality, frequency)?

# Participant description items

- Were participant ages adequately described?
- Were participant education levels adequately described?
- Were participant diagnoses adequately described?
- Were participant medications adequately described?
- Were participant cognitive abilities (e.g., working memory, executive function, sustained attention, divided attention, alternating attention, etc.) adequately described?
- Were participant communication abilities (e.g. speech, receptive and/or expressive language) adequately described?
- Were participant visual abilities (e.g., acuity, oculomotor, disorders) adequately described?
- Were participant auditory abilities adequately described?
- Were participant motor abilities adequately described?
- Were participant literacy skills adequately described?
- Did the study specify whether participants had previous BCI experience?
